# Structure-activity relationships for unit C pyridyl analogues of the tuberculosis drug bedaquiline

**DOI:** 10.1016/j.bmc.2019.02.025

**Published:** 2019-04-01

**Authors:** Adrian Blaser, Hamish S. Sutherland, Amy S.T. Tong, Peter J. Choi, Daniel Conole, Scott G. Franzblau, Christopher B. Cooper, Anna M. Upton, Manisha Lotlikar, William A. Denny, Brian D. Palmer

**Affiliations:** a Auckland Cancer Society Research Centre, School of Medical Sciences, University of Auckland, Private Bag 92019, Auckland 1142, New Zealand; b Maurice Wilkins Centre, University of Auckland, Private Bag 92019, Auckland 1142, New Zealand; c Institute for Tuberculosis Research, College of Pharmacy, University of Illinois at Chicago, 833 South Wood Street, Chicago, IL 60612, USA; d Global Alliance for TB Drug Development, 40 Wall Street, NY 10005, USA

**Keywords:** Bedaquiline, Bedaquiline analogues, Lipophilicity, Tuberculosis, Drug development

## Abstract

The ATP-synthase inhibitor bedaquiline is effective against drug-resistant tuberculosis but is extremely lipophilic (clogP 7.25) with a very long plasma half-life. Additionally, inhibition of potassium current through the cardiac hERG channel by bedaquiline, is associated with prolongation of the QT interval, necessitating cardiovascular monitoring. Analogues were prepared where the naphthalene C-unit was replaced with substituted pyridines to produce compounds with reduced lipophilicity, anticipating a reduction in half-life. While there was a direct correlation between *in vitro* inhibitory activity against *M. tuberculosis* (MIC_90_) and compound lipophilicity, potency only fell off sharply below a clogP of about 4.0, providing a useful lower bound for analogue design. The bulk of the compounds remained potent inhibitors of the hERG potassium channel, with notable exceptions where IC_50_ values were at least 5-fold higher than that of bedaquiline. Many of the compounds had desirably higher rates of clearance than bedaquiline, but this was associated with lower plasma exposures in mice, and similar or higher MICs resulted in lower AUC/MIC ratios than bedaquiline for most compounds. The two compounds with lower potency against hERG exhibited similar clearance to bedaquiline and excellent efficacy *in vivo*, suggesting further exploration of C-ring pyridyls is worthwhile.

## Graphical abstract

**Figure:**
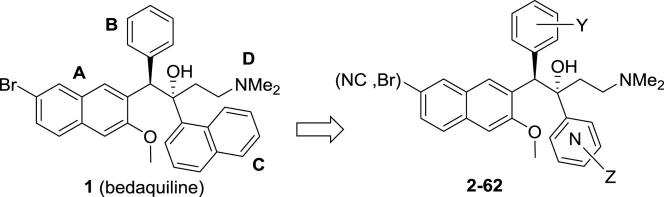

## 1 Introduction

**Fig. 1:**
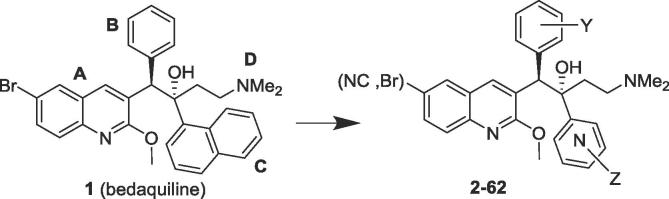
Bedaquiline and C-unit pyridyl analogues.

Bedaquiline (TMC207, Sirturo, Janssen Pharmaceuticals; Fig. 1; **1**) is an exciting new drug for tuberculosis, with a novel mechanism of inhibition of the mycobacterial ATP synthase.^1^ Of particular interest, it has demonstrated clinical efficacy against multidrug-resistant TB, where treatment options are limited.^2^ Potential limitations of **1** include very high lipophilicity (a calculated clogP of 7.25),^3^ with the attendant risks of ultra-long half-life,^4^ phospholipidosis,^5^ and drug insolubility. In attempts to develop analogues with similar or improved potency against *Mycobacterium tuberculosis*, but with lower lipophilicity than bedaquiline, anticipating a desirably shorter half-life, we have recently reported on the beneficial effects of replacing the lipophilic 6-Br group on the A-unit quinoline ring with a more hydrophilic cyano group,^6^ replacing the B-unit phenyl group with monoheterocycles,^7^ and replacing the very lipophilic C-unit naphthalene with a series of less lipophilic bicyclic heterocycles.^8^ In the present work we take the latter theme further by looking at a range of even less lipophilic pyridine C-units, with a further comparison of paired Br/CN derivatives. A recent paper^9^ on the 1.7 Å resolution crystal structure of **1** bound to the c subunit of the ATP synthase Fo of the genetically-similar mycobacterium *M. phlei* (83.7%), found that the quinoline and naphthalene units appear to play a role in positioning the dimethylaminoethyl side chain in the ion-binding site of the enzyme. Following on from this, it was of interest to see whether analogues with smaller, more hydrophilic, monocyclic pyridyl C-units would be as effective at binding. The only other study^1^ on non-naphthalene analogues of **1** showed that 2-fluoro- and 2,5-difluorophenyl compounds had MIC_90_ values (in *Mycobacterium smegmatis*) comparable to that of **1**, but pyridyl analogues were not examined.

## 2 Results and discussion

### 2.1 Chemistry

**Scheme 1:**
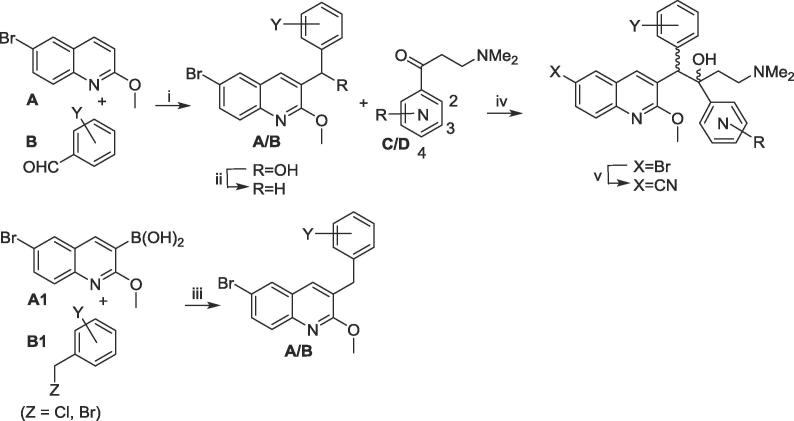
General synthesis of bedaquiline analogues. Reagents and conditions: (i) LiTMP, THF, −75 °C, 1.5 h then the appropriate aldehyde **B**, −75 °C, 4 h; (ii) Et_3_SiH, TFA, DCM; (iii) Cs_2_CO_3_, Pd(PPh_3_)_4_, PhMe/DMF, 110 °C (sealed tube), 5 h; (iv) LDA, THF, −75 °C, 1.5 h then the appropriate ketone **C/D**, then HOAc; (v) Zn/Zn(CN)_2_, Pd_2_(dba)_3_/P(o-tol)_3_, DMF, 50 °C, then separation of the diastereomers by SFC HPLC.

The bedaquiline analogues **2**–**62** were synthesized from appropriate benzylquinoline A/B-units and 3-(dimethylamino)-1-arylpropan-1-one C/d-units, as previously reported,^3^, ^6^ and as shown in Scheme 1.

**Scheme 2:**
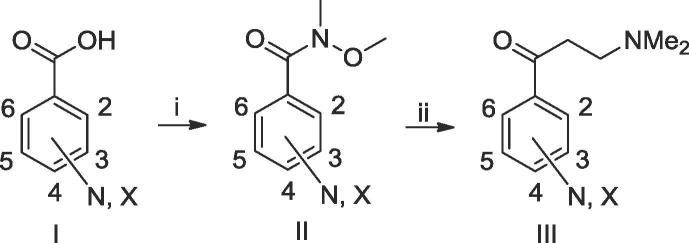
Synthesis of new pyridyl 3-(dimethylamino)propan-1-one C/D units. Reagents and conditions: (i) COCl_2_, cat. DMF, DCM, then MeNH(OMe). HCl, pyridine; (ii) vinylMgBr, then Me_2_NH, THF.

Lithium tetramethylpiperidide (LiTMP) mediated condensation of quinoline **A** and substituted benzaldehydes **B** gave the intermediate alcohols, which were deoxygenated by triethylsilane under acid conditions to give the corresponding **A/B** units. Alternatively, palladium mediated coupling of the boronic acid **A1** with an appropriately substituted benzyl halide or (halomethyl)pyridine **B1** gave the **A/B** units, all of which have been reported previously.^3^, ^6^, ^7^ The Mannich bases required for the **C/D** units of the final compounds were prepared from the appropriate commercial carboxypyridines as shown in Scheme 2 and Table 1. Lithiation of the **A/B** subunit with lithium diisopropylamide (LDA) and subsequent reaction with the **C/D** subunit gave the bromo bedaquiline analogues. The cyano derivatives were synthesised from their corresponding bromo derivatives by palladium-catalysed cyanation.

**Table 1:** New Mannich base C/D units prepared via Scheme 2.

Class	N	X^a^	% yields I/II/III
A	2-	3-Me	95/97
B	2-	5-Me	31/99
C	2-	6-Me	46/99
D	2-	3,5-diMe	88/98
E	2-	4,6-diMe	23/99
F	2-	3-OMe	92/95
G	2-	5-OMe	72/99
H	3-	2-Me	83/75
I	3-	4-Me	95/97
J	3-	5-Me	87/93
K	3-	2,4-diMe	66/99
L	3-	4,5-diMe	92/98
M	3-	4,6-diMe	93/99
N	3-	2-OMe	78/99
O	3-	4-OMe	83/99
P	3-	2,5-diOMe	61/90
Q	3-	4,5-diOMe	85/83
R	3-	2,4,5-triOMe	94/92
S	4-	2-Me	90/31
T	4-	3-Me	79/99
U	4-	3,5-diMe	62/99
V	4-	3,5-diEt	82/99
W	4-	2,5-diOMe	41/67
X	4-	3-OMe, 5-*^n^*Pr	97/99
X	4-	3-OMe, 5-^i^Pr	91//99

a For clarity, substituent numbering is consistent with that in Table 2, and is not necessarily IUPAC.

Bedaquiline (1) and C-unit pyridyl analogues **2**–**62** were evaluated for their ability to inhibit bacterial growth (measured as MIC_90_ values in µg/mL against *M. tb* (strain H37Rv)) under both replicating (MABA)^10^ or non-replicating (LORA)^11^ conditions. In these assays, **1** is a potent inhibitor, with MICs of 0.08 and 0.12 µg/mL, respectively. Replacement of the naphthalene C-unit of **1** with various pyridyls produced analogues of considerably lower lipophilicity, with clogP values ranging from 6.89 down to 2.98.

### 2.2 Structure-activity relationships

This work explored SAR for much less lipophilic analogues of bedaquiline, with clogP values between 6.89 and 2.98 (0.36 to 4.27 log units less lipophilic than bedaquiline). Overall, that resulted in compounds that are slightly less potent than bedaquiline (1) against both replicating (MABA) and non-replicating (LORA) *M.tb* in culture (Table 2). As expected from earlier SAR work on this series,^6^, ^7^, ^8^ there was a good overall correlation between the MABA and LORA MIC_90_ values for the compounds:

(1) $$log\left(\mathrm{MABA}\right)=0.98*log\left(\mathrm{LORA}\right)-0.08$$

$$n=58,\;R=0.85,\;p<0.001,\;F_{2,56}=144$$

**Table 2:** Structures and biological activity of bedaquiline C-unit pyridyl analogues.

**Figure:**
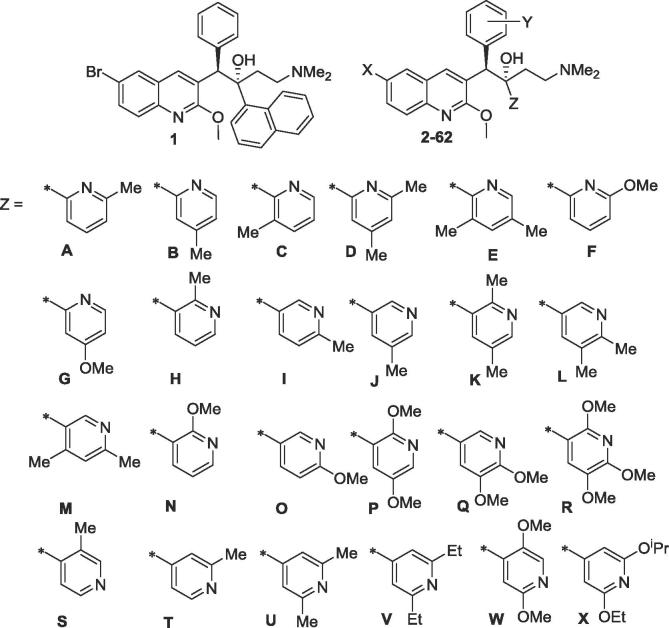

No	X	Y	Z	Yield^a^	MIC_90_^b^	clogP^c^	
				AB/CD	MABA	LORA	
**1**					0.08	0.12	7.25
**2**	Br	2,3-diOMe	A: 2-aza, 3-Me	69	0.40	0.13	4.34
**3**	CN	2,3-diOMe	A: 2-aza, 3-Me	45^d^	4.1	1.9	2.98
**4**	Br	2-F, 3-OMe	A; 2-aza, 3-Me	79	0.29	2.0	5.55
**5**	CN	2-F, 3-OMe	A: 2-aza, 3-Me	93^d^	1.1	1.8	4.26
**6**	Br	2-F, 3-OMe	B: 2-aza, 5-Me	56	0.6	2.1	5.08
**7**	Br	2-F, 3-OMe	C: 2-aza, 6-Me	77	0.19	0.40	5.03
**8**	CN	2-F, 3-OMe	C: 2-aza, 6-Me	83^d^	3.0	3.5	3.67
**9**	Br	2,3-diOMe	D: 2-aza, 3,5-diMe	56	0.28	0.30	4.84
**10**	CN	2,3-diOMe	D: 2-aza, 3,5-diMe	66^d^	0.54	0.62	3.48
**11**	Br	2-F, 3-OMe	D: 2-aza, 3,5-diMe	65	0.14	0.11	5.58
**12**	CN	2-F, 3-OMe	D: 2-aza, 3,5-diMe	50^d^	2.3	2.8	4.22
**13**	Br	2-F, 3-OMe	E: 2-aza, 4,6-diMe	43	0.30	0.46	5.53
**14**	CN	3-Me	F: 2-aza, 3-OMe	36^d^	0.60	0.29	4.54
**15**	Br	2,3-OCH_2_O-	F: 2-aza, 3-OMe	46	0.54	0.58	5.36
**16**	CN	2,3-OCH_2_O-	F: 2-aza, 3-OMe	85	1.2	ND	4.01
**17**	Br	2-F, 3-OMe	G: 2-aza, 5-OMe	56	1.2	0.61	5.00
**18**	CN	2-F, 3-OMe	G: 2-aza, 5-OMe	78^d^	2.3	2.2	3.65
**19**	Br	3-Me	H: 3-aza, 2-Me	26	0.15	0.25	5.54
**20**	Br	2,3-diOMe	H: 3-aza, 2-Me	46	0.30	0.59	4.29
**21**	Br	2,3-OCH_2_O-	H: 3-aza, 2-Me	23	0.45	0.56	4.99
**22**	Br	2-F, 3-OMe	I: 3-aza, 4-Me	59	0.30	0.28	5.55
**23**	CN	2-F, 3-OMe	I: 3-aza, 4-Me	93^d^	4.7	>5	3.72
**24**	Br	2,3-diOMe	I: 3-aza, 4-Me	59	0.60	0.51	4.34
**25**	CN	2,3-diOMe	I: 3-aza, 4-Me	54^d^	>5	>5	2.98
**26**	Br	3-F	J: 3-aza, 5-Me	61	0.28	0.08	5.22
**27**	CN	3-F	J: 3-aza, 5-Me	62^d^	1.2	0.98	3.87
**28**	Br	2,3-diOMe	J: 3-aza, 5-Me	67	0.30	0.57	4.34
**29**	CN	2,3-diOMe	J: 3-aza, 5-Me	68^d^	4.2	3.8	2.98
**30**	Br	2-F, 3-OMe	J: 3-aza, 5-Me	22	0.16	0.20	5.08
**31**	CN	2-F, 3-OMe	J: 3-aza, 5-Me	74^d^	3.3	2.5	3.72
**32**	Br	2-F, 3-OMe	K: 3-aza, 2,4-diMe	22	0.07	0.23	5.53
**33**	Br	2–3-diOMe	K: 3-aza, 2,4-diMe	29	0.07	0.11	4.84
**34**	Br	2–3-diOMe	L: 3-aza, 4,5-diMe	70	0.07	0.14	4.80
**35**	Br	2-F, 3-OMe	L: 3-aza, 4,5-diMe	52	0.31	0.29	5.53
**36**	CN	2-F, 3-OMe	L: 3-aza, 4,5-diMe	70^d^	2.4	3.3	4.17
**37**	Br	2-F, 3-OMe	M: 3-aza, 4,6-diMe	14	0.53	0.29	5.53
**38**	Br	2,3-diOMe	M: 3-aza, 4,6-diMe	32	0.02	0.24	4.79
**39**	Br	3-Me	N: 3-aza, 2-OMe	50	0.13	0.14	5.90
**40**	CN	3-Me	N: 3-aza, 2-OMe	82	0.52	0.76	4.04
**41**	Br	2,3-OCH_2_O-	N: 3-aza, 2-OMe	53	0.66	1.1	5.36
**42**	CN	2,3-OCH_2_O-	N: 3-aza, 2-OMe	78^d^	2.1	ND	4.01
**43**	Br	2,3-diOMe	O: 3-aza, 4-OMe	57	0.28	0.54	4.66
**44**	CN	2,3-diOMe	O: 3-aza, 4-OMe	90^d^	2.1	2.1	3.30
**45**	Br	2-F, 3-OMe	O: 3-aza, 4-OMe	33	0.12	0.08	5.40
**46**	Br	2-F, 3-OMe	P: 3-aza, 2,5-diOMe	29	0.09	0.04	5.80
**47**	Br	2-F, 3-OMe	Q: 3-aza, 4,5-diOMe	30	0.07	0.11	5.45
**48**	Br	4-aza, 2,3-diOMe	Q: 3-aza, 4,5-diOMe	83	0.06	0.45	4.42
**49**	Br	4-aza, 2,3-diOMe	R: 3-aza, 2,4,5-triOMe	42	0.57	1.4	4.80
**50**	Br	2-F, 3-OMe	S: 4-aza, 2-Me	54	1.0	1.1	5.03
**51**	Br	2-F, 3-OMe	T: 4-aza, 3-Me	47	0.47	0.58	5.08
**52**	CN	2-F, 3-OMe	T: 4-aza, 3-Me	82^d^	>5	3.8	3.72
**53**	Br	3-Me	U: 4-aza, 3,5-diMe	20	0.11	0.49	6.08
**54**	Br	2-F, 3-OMe	U: 4-aza, 3,5-diMe	46	0.11	0.30	5.58
**55**	CN	2-F, 3-OMe	U: 4-aza, 3,5-diMe	59^d^	0.60	0.91	4.22
**56**	Br	2,3-diOMe	U: 4-aza, 3,5-diMe	63	0.04	0.07	4.79
**57**	CN	2,3-diOMe	U: 4-aza, 3,5-diMe	75^d^	0.24	0.61	4.01
**58**	Br	2-F, 3-OMe	V: 4-aza, 3,5-diEt	45	0.03	0.21	7.10
**59**	CN	2-F 3-OMe	V: 4-aza, 3,5-diEt	67	0.19	0.28	5.81
**60**	Br	2-F, 3-OMe	W: 4-aza, 2,5-diOMe	55	0.11	0.12	5.80
**61**	Br	4-aza, 2,3-diOMe	X: 4-aza, 3-OMe, 5-O*^n^*Pr	45	0.01		
	0.06	5.83					
**62**	Br	4-aza, 3,5-diOMe	X: 4-aza, 3-OEt, 5-O^i^Pr	59	<0.02	<0.02	6.89

a Yields in the AB/CB coupling step to give bedaquiline analogues (as racemic mixtures; these were then separated by super-critical fluid HPLC at BioDuro LLC (Beijing) and the desired *RS*,*SR* diastereomers (depicted) were evaluated).

b MIC_90_ (µg/mL); minimum inhibitory concentration for 90% inhibition of growth of *M.tb* strain H37Rv, determined under aerobic (replicating; MABA) (ref. 10) or non-replicating (LORA) (Ref. 11) conditions, determined at the Institute for Tuberculosis Research, University of Illinois at Chicago.

c clogP calculated by ChemDraw Ultra v12.0.2. (CambridgeSoft).

d Yields for the Br/CN conversion.

Also, as expected, there was a reasonable correlation between inhibitory activity (illustrated here for MABA data) and compound lipophilicity, with the more lipophilic compounds being more potent; an overall trend repeatedly seen in other classes of TB drugs.^12^, ^13^

(2) logMABA=-0.50∗clogP+1.97

$$n=58,\;R=0.71,\;P<0.001,\;F_{2,56}=55$$

However, this overall correlation masks a trend seen to some extent previously,^8^ that for this general class of compound, MIC_90_ potency falls off quite sharply for compounds (e.g., **3**, **8**, **16**, **18**, **23**, **25**, **27**, **29**, **31**, **42**, **44**, **52**) of lower lipophilicity (clogP values around 4 or below).

Of the many different C-units employed in this study, there were nine (A, D, I, J, K, L, M, O, and U) that had identical A-units (6-Br) and examples of both 2-F, 3-OMe, and 2,3-diOMe B-units, allowing an analysis of any effects of these B-unit changes on activity. However, the averaged MIC_90_ values for each series was identical (0.23 µg/mL), suggesting these B-unit changes have minimal effects on activity.

Representative compounds in the series were evaluated for a range of ADME and toxicological properties *in vitro* (Table 3). Compounds were tested for cytotoxicity in Vero green monkey-derived epithelial kidney cells,^14^ and for inhibition of CYP 3A4, the major metabolising enzyme for **1**.^15^ All tested compounds had IC_50_s > 10 µg/mL in the Vero assay, except for compound **38**, where this was not measured (the value for **1** in repeat assays was between 4 and 16 µg/mL). All compounds also had IC_50_s > 10 µM for CYP3A4 inhibition (bedaquiline IC_50_ > 40 µM), except for compound **15**, which had an IC_50_ of 7.6 µM, and compounds **4**, **27**, **33**, **38**, **46**, **48**, **49**, **57**–**59**, **60**–**62,** which were not tested in this assay.

**Table 3:** Additional biological data for selected representative compounds of Table 2.

No.	*in vitro* parameters			*in vivo* parameters						
	hERG^a^	HCl_int_^b^	MCl_int_^c^	IV Cl^d^	V_z_^e^	AUC^f^	F^g^	CFU^h^	CFU^i^	AUC/MIC_90_ MABA
**1**	1.6	3	7.3	7	22	20.9	56			261
**4**	34%/3	10	39				ND			
**15**	0.24	8	25	13	10	5.60	45	0.3	4.9	10.4
**19**	26%/3	35	71	59	33	0.40	10			
**21**	13%/3	71	51	44	21	0.82	19			
**22**	17%/3	24	99	67	10	0.25	8			
**27**	42%/1	122	249	ND	ND	ND	ND			
**33**	1.1	7	17	79	77	1.39	72	3.5	5.0	19.9
**38**	3.9	13	46	28	23	2.08	34	0.7	>4.5	104
**39**	0.45	10	20	ND	ND	2.51	ND	0.6	4.9	19.3
**40**	0.30	11	53	48	6.6	1.17	33	0.5	6.2	2.25
**41**	26%/3	36	19	27	20	3.78	46	0.3	5.5	5.73
**46**	2.9	7	53	ND	ND	ND	ND			
**48**	3.6	5	59	19	6.0	4.28	48	0.9	>5	71.3
**49**	2.7	5	7	10	12	9.13	52			
**55**	37%/3	12	51	130	53	0.35	25			
**57**	5.2	7	7	71	82	1.48	54	1.9	5.8	6.17
**58**	63%/3	8	20	ND	ND	ND	ND			
**59**	48%/1	10	27	ND	ND	ND	ND			
**60**	1.7	7	25	ND	ND	ND	ND			
**61**	7.8	8	7	7	44	10.9	50	5	5.5	1090
**62**	>10	1	2.3	3	20	33.0	58	4.5	>5	1650

a Inhibition of hERG (IC_50_ in µM or % inhibition at 1 or 3 µM in the manual assay or 3 µM in the (less accurate) QPatch assay.

b Clearance of compound by human liver microsomes (μL/min/mg protein).

c Clearance of compound by mouse liver microsomes (µL/min/mg protein).

d IV clearance, mouse (mL/min/kg).

e IV apparent volume of distribution during terminal phase, mouse (L/kg).

f Exposure; mouse AUC_inf_ (µg*h/mL); ^g^Oral bioavailability in mice (%).

h,i Log reduction in colony-forming units (CFU) of new compounds^h^ compared to **1**^i^ run in the same assay, when dosed at 20 mg/kg daily in mice for 12 days, beginning 10 days after *M.tb* inoculation via the aerosol route.

Since bedaquiline’s relatively potent inhibition of the hERG potassium channel (IC_50_ 1.6 µM^14^ (see Table 3) has been associated with QTc prolongation observed in the clinic (cardiovascular toxicity), the analogue compounds were also evaluated for inhibition of hERG potassium current *in vitro*. This was determined either by a full (5-concentration) dose–response study resulting in an IC_50_, or by determining % inhibition of channel current at 0.3 and 1 µM, using the same manual patch clamp electrophysiology assay setup. While several of the compounds (**38**, **46**, **48**, **49**, **57**, **61**, **62**) were less potent inhibitors of this channel than **1**, several others (e.g., **15**, **33**, **39**, **40**, **59**, **60**) were similar or considerably more potent. Lipophilicity did not seem to be a factor, with both **1** (clogP 7.25) and compound **27** (clogP 3.87) being similarly potent against the hERG channel. Compounds **61** and **62**, bearing 4-aza, 3,5-dialkoxy C-units, were notable for their much lower hERG potencies (IC_50_ values around or >10 µM), suggesting this motif as a promising one for mitigation of hERG risk.

One of the cited issues with **1** is its very long terminal half-life in humans.^16^ This can result in undesirable levels of accumulation of drug in tissues. Table 3 also shows *in vitro* clearance rates of the analogues in human and mouse microsomes as well as the *in vivo* clearance observed in mice. All of the pyridyl compounds had (desirably) higher rates of clearance *in vitro* than **1**, with several (**4**, **19**, **21**, **22**, **27**) possibly being cleared too rapidly. This was also true for those pyridyl compounds evaluated *in vivo*, with several demonstrating clearance >50 mL/min/kg (**19, 22, 33, 55, 57**) compared to 7 mL/min/kg for bedaquiline.

Several analogues were also evaluated for oral bioavailability in mice, and while being less bioavailable than the very lipophilic **1**, still had acceptable values. As seen in Table 3, in general, higher *in vivo* clearance values predicted lower *in vivo* AUC and lower %F, suggesting oral bioavailability for these compounds is primarily driven by clearance as opposed to absorption. The volume of distribution for these compounds was at least 6 L/kg in all cases (22 L/kg for bedaquiline) and varied among the compounds (see Table 3), but did not appear to relate to clogP. All compounds tested demonstrated human plasma protein binding of >99.9% (data not shown).

Ten representative compounds with a range of MABA MICs, volume of distribution and AUC values were evaluated for efficacy in a mouse model of TB. In this model, the infected mice are dosed daily with 20 mg/kg of the test compound for 12 days, beginning 10 days after *M.tb* infection. Efficacy is measured by the log reduction in colony-forming units (CFU) recovered from the lungs, compared to **1** as a positive control in the same assay. The studies sought to identify compounds that demonstrated similar efficacy to **1** at the same dose (a lung CFU reduction of similar magnitude).

Of the compounds evaluated *in vivo*, only compounds **61** and **62** effected a lung CFU reduction similar to that of **1,** at the same dose. These compounds were also the only analogues with both AUCs and MABA MICs comparable to that of **1**. These two compounds had the highest AUC/MIC ratios of the novel analogues tested, and were the only compounds evaluated for efficacy with AUC/MIC similar or higher than AUC/MIC of **1**. The resultant efficacy is consistent with the results of a detailed previous study that identified plasma AUC/MIC as the driver of the *in vivo* efficacy of **1** in a similar mouse model of TB.^16^ Of interest, compounds **33** and **57** demonstrated efficacy superior to that of the other analogues (except for **61** and **62**), despite low AUC/MIC ratios. It is notable that these compounds demonstrated extremely high volumes of distribution, suggesting especially high tissue levels. It is also possible that these two compounds generate active metabolites *in vivo* that contribute to efficacy; bedaquiline is known to generate an active metabolite that contributes to efficacy in mice.^17^ Further studies will be needed to determine whether active metabolites are also present in mice, following oral dosing of the analogues described here.

## 3 Conclusions

In this study we prepared and evaluated a set of much more polar analogues of bedaquiline (1), by replacing the naphthalene C-unit with a series of substituted pyridyls. As shown previously, the potency (MIC_90_ values) of these compounds against *M.tb* in culture correlated positively with high lipophilicity, but this study was able to set a lower bound to lipophilicity (clogP about 4), below which *in vitro* potency decreased sharply. Encouragingly, many of the C-pyridyl analogues proved to be slightly less potent inhibitors of the hERG potassium channel than bedaquiline itself, and two examples (**61** and **62**) demonstrated IC_50_ values of 7.8 and >10 µM.

This set demonstrated varying *in vitro* and *in vivo* clearance values, with some compounds demonstrating clearance values desirably higher than those of bedaquiline. However, in part due to higher clearance values and resultant lower AUCs, this set of compounds, in general, had poor *in vivo* activity against murine TB compared to bedaquiline given at the same dose. The exceptions were compounds **61** and **62**, which had comparable *in vivo* activity to **1** and were the only tested compounds with AUC/MIC ratios at least as high as that of **1**. Other tested compounds had lower AUCs or higher MICs or both, compared to bedaquiline, with lower AUC/MIC ratios as a result. The observation that higher AUC/MIC results in superior efficacy for this class is consistent with previous studies of bedaquiline that indicated AUC/MIC as the driver of efficacy for that compound against murine TB.

Thus, while higher clearance than bedaquiline is a goal for this program of work, it appears that this must be accompanied by lower MICs than 1 in order to maintain the AUC/MIC ratio required to produce similar efficacy to bedaquiline, at the same dose. Although the present work did not identify a compound with lower MIC and higher clearance than bedaquiline, compounds **61** and **62**, which demonstrated similar efficacy to bedaquiline with similar clearance values, also showed considerably less potent hERG inhibition, suggesting that further exploration of pyridyl-based C units might be fruitful.

## 4 Experimental

### 4.1 Chemistry

Final products were analysed by reverse-phase HPLC (Alltima C18 5 µm column, 150 × 3.2 mm; Alltech Associated, Inc., Deerfield, IL) using an Agilent HP1100 equipped with a diode-array detector. Mobile phases were gradients of 80% CH_3_CN/20% H_2_O (v/v) in 45 mM NH_4_HCO_2_ at pH 3.5 and 0.5 mL/min. Purity was determined by monitoring at 330 ± 50 nm and was ≥95% for all final products. Melting points were determined on an Electrothermal 9100 melting point apparatus. NMR spectra were obtained on a Bruker Avance 400 spectrometer at 400 MHz for ^1^H. Low-resolution atmospheric pressure chemical ionization (APCI) mass spectra were measured for organic solutions on a ThermoFinnigan Surveyor MSQ mass spectrometer, connected to a Gilson autosampler.

#### 4.1.1 New C/D units (Table 1)

##### 4.1.1.1 3-(Dimethylamino)-1-(6-methylpyridin-2-yl)propan-1-one (class A)

Oxalyl chloride (1.85 mL, 21.88 mmol) was added to a suspension of 6-methylpicolinic acid (2.50 g, 18.23 mmol) in DCM (75 mL, anhydrous) and DMF (1.3 mmol) at 20 °C. The mixture was stirred at 20 °C for 1 h to give a colorless solution which was cooled to 0 °C. *N,O*-Dimethylhydroxylamine hydrochloride (1.95 g, 20.1 mmol) and pyridine (3.25 mL, 40.11 mmol) were added sequentially and the mixture was stirred at 20 °C for 18 h, then partitioned between EtOAc and sat. aq. NaHCO_3_. Column chromatography with hexanes:EtOAc 1:1 gave *N*-methoxy-*N*,6-dimethylpicolinamide as an oil (2.68 g, 82%). ^1^H NMR (CDCl_3_) *δ* 7.66 (t, *J* = 7.8 Hz, 1H), 7.44 (s, 1H), 7.21 (dd, *J* = 7.6, 0.4 Hz), 3.76 (s, 3H), 3.39 (s, 3H), 2.59 (s, 3H). Found: [M+H] = 181.1.

Vinylmagnesium bromide solution in THF (1 M, 45 mL, 44.6 mmol) was added to a solution of *N*-methoxy-N,6-dimethylpicolinamide (2.68 g, 14.87 mmol) in THF (130 mL, dist. Na) at 0 °C, the brown solution was warmed to 20 °C for 1 h then dimethylamine in THF (2 N, 45 mL, 89.2 mmol) and water (23 mL) were added. The solution was stirred at 20 °C for 1 h, then partitioned between EtOAc and water. The solution was dried and evaporated to give 3-(dimethylamino)-1-(6-methylpyridin-2-yl)propan-1-one as a yellow oil (2.78 g, 97%). ^1^H NMR (CDCl_3_) *δ* 7.83 (dd, *J* = 7.7, 0.4 Hz, 1H), 7.69 (t, *J* = 7.7 Hz, 1H), 7.31 (dd, *J* = 7.7, 0.4 Hz, 1H), 3.40 (t, *J* = 7.2 Hz, 2H), 2.77 (t, *J* = 7.2 Hz, 2H), 2.61 (s, 3H), 2.29 (s, 6H). Found: [M+H] = 192.8.

##### 4.1.1.2 3-(Dimethylamino)-1-(4-methylpyridin-2-yl)propan-1-one (Class B)

This was prepared similarly from 4-methylpicolinic acid to give *N*-methoxy-*N*,4-dimethylpicolinamide in 31% yield. ^1^H NMR (CDCl_3_) *δ* 8.46 (d, *J* = 5.0 Hz, 1H), 7.49 (br, 1H), 7.17 (dd, *J* = 5.0, 0.8 Hz, 1H), 3.77 (br s, 3H), 3.40 (br s, 3H), 2.40 (s, 3H). Found: [M+H] = 181.1.

This Weinreb amide was similarly converted into 3-(dimethylamino)-1-(4-methylpyridin-2-yl)propan-1-one in 99% yield. ^1^H NMR (CDCl_3_) *δ* 8.53 (d, *J* = 4.9 Hz, 1H), 7.86 (m, 1H), 7.28 (m, 1H), 3.39 (t, *J* = 7.2 Hz, 2H), 2.77 (t, *J* = 7.2 Hz, 2H), 2.42 (s, 3H), 2.84 (s, 6H). Found: [M+H] = 193.0.

##### 4.1.1.3 3-(Dimethylamino)-1-(3-methylpyridin-2-yl)propan-1-one class C)

This was prepared similarly from 3-methylpicolinic acid to give *N*-methoxy-*N*,3-dimethylpicolinamide in 46% yield. ^1^H NMR (CDCl_3_) *δ* 8.44 (dd, *J* = 4.7, 0.8 Hz, 1H), 7.56 (d, *J* = 7.6 Hz, 1H), 7.25 (dd, *J* = 7.7, 4.8 Hz, 1H), 3.56 (s, 3H), 3.40 (s, 3H), 2.35 (s, 3H). Found: [M+H] = 181.0.

This Weinreb amide was similarly converted into 3-(dimethylamino)-1-(3-methylpyridin-2-yl)propan-1-one in 99% yield. ^1^H NMR (CDCl_3_) *δ* 8.50 (dd, *J* = 4.6, 1.1 Hz, 1H), 7.57 (dq, *J* = 7.8, 0.7 Hz, 1H), 7.31 (dd, *J* = 7.7, 4.6 Hz, 1H), 3.36 (t, *J* = 7.2 Hz, 2H), 2.73 (t, *J* = 7.2 Hz, 2H), 2.56 (s, 3H), 2.27 (s, 6H). Found: [M+H] = 192.8.

##### 4.1.1.4 3-(Dimethylamino)-1-(4,6-dimethylpyridin-2-yl)propan-1-one (Class D)

This was prepared similarly from 4,6-dimethylpicolinic acid to give *N*-methoxy-*N*,4,6-trimethylpicolinamide in 88% yield. ^1^H NMR (CDCl_3_) *δ* 7.26 (s, 1H), 7.04 (s, 1H), 3.77 (br s, 1H), 3.38 (br s, 3H), 2.54 (s, 3H), 2.34 (s, 3H). Found [M+H] = 195.0.

This Weinreb amide was similarly converted into 3-(dimethylamino)-1-(4,6-dimethylpyridin-2-yl)propan-1-one in 98% yield. ^1^H NMR (CDCl_3_) *δ* 7.64 (s, 1H), 7.11 (s, 1H), 3.36 (t, *J* = 7.1 Hz, 2H), 2.74 (t, *J* = 7.1 Hz, 2H), 2.54 (s, 3H), 2.34 (s, 3H), 2.27 (s, 6H). Found: [M+H] = 207.2.

##### 4.1.1.5 3-(Dimethylamino)-1-(3,5-dimethylpyridin-2-yl)propan-1-one (Class E)

This was prepared similarly from 3,5-dimethylpicolinic acid to give *N*-methoxy-*N*,3,5-trimethylpicolinamide in 23% yield. ^1^H NMR (CDCl_3_) *δ* 8.25 (d, *J* = 0.6 Hz, 1H), 7.36 (s, 1H), 3.56 (s, 3H), 3.37 (s, 3H), 2.33 (s, 3H), 2.31 (s, 3H). Found: [M+H] = 195.1.

This Weinreb amide was similarly converted into 3-(dimethylamino)-1-(3,5-dimethylpyridin-2-yl)propan-1-one in 99% yield. ^1^H NMR (CDCl_3_) *δ* 8.32 (dd, *J* = 1.3, 0.5 Hz, 1H), 7.37 (dd, *J* = 1.3, 0.7 Hz, 1H), 3.35 (t, *J* = 7.2 Hz, 2H), 2.73 (t, *J* = 7.2 Hz, 2H), 2.54 (s, 3H), 2.36 (s, 3H), 2.27 (s, 6H). Found: [M+H] = 207.2.

##### 4.1.1.6 3-(Dimethylamino)-1-(6-methoxypyridin-2-yl)propan-1-one (Class F)

This was prepared similarly from 6-methoxypicolinic acid to give *N*,6-dimethoxy-*N*-methylpicolinamide in 92% yield. ^1^H NMR (CD_3_SOCD_3_) *δ* 7.86 (dd, *J* = 8.3, 7.3 Hz, 1H), 7.66 (dd, *J* = 7.3, 0.7 Hz, 1H), 7.05 (dd, *J* = 8.3, 0.7 Hz, 1H), 3.90 (s, 3H), 3.28 (bs, 3H). Found: [M+H] = 197.0.

The Weinreb amide was similarly converted into 3-(dimethylamino)-1-(6-methoxypyridin-2-yl)propan-1-one in 69% yield. ^1^H NMR (CDCl_3_) *δ* 7.69 (t, *J* = 7.3 Hz, 1H), 7.64 (dd, *J* = 7.3, 1.0 Hz, 1H), 6.93 (dd, *J* = 8.0, 1.0 Hz, 1H), 3.99 (s, 3H), 3.36 (t, *J* = 7.2 Hz, 2H), 2.77 (t, *J* = 7.2 Hz, 2H), 2.29 (s, 3H). Found: [M+H] = 209.1.

##### 4.1.1.7 3-(Dimethylamino)-1-(4-methoxypyridin-2-yl)propan-1-one (Class G)

This was prepared similarly from 4-methoxypicolinic acid to give *N*,4-dimethoxy-*N*-methylpicolinamide in 72% yield. ^1^H NMR (CDCl_3_) *δ* 8.42 (d, *J* = 5.7 Hz, 1H), 7.19 (s, 1H), 6.87 (dd, *J* = 5.7, 2.6 Hz, 1H), 3.89 (s, 3H), 3.77 (s, 3H), 3.41 (s, 3H). Found: [M+H] = 197.1.

This Weinreb amide was similarly converted into 3-(dimethylamino)-1-(4-methoxypyridin-2-yl)propan-1-one in 99% yield. ^1^H NMR (CDCl_3_) *δ* 8.48 (d, *J* = 5.6 Hz, 1H), 7.57 (d, *J* = 2.6 Hz, 1H), 6.97 (dd, *J* = 5.6, 2.6 Hz, 1H), 3.90 (s, 3H), 3.38 (t, *J* = 7.2 Hz, 2H), 2.77 (t, *J* = 7.2 Hz, 2H), 2.28 (s, 6H). Found: [M+H] = 209.0.

##### 4.1.1.8 3-(Dimethylamino)-1-(2-methylpyridin-3-yl)propan-1-one (Class H)

This was prepared similarly from 2-methylnicotinic acid to give *N*-methoxy-*N*,2-dimethylnicotinamide in 83% yield. ^1^H NMR (CDCl_3_) *δ* 8.55 (dd, *J* = 4.9, 1.8 Hz, 1H), 7.60 (dd, *J* = 7.7, 1.8 Hz, 1H), 7.16 (dd, *J* = 7.7, 4.9 Hz, 1H), 3.46 (br s, 3H), 3.36 (br s, 3H), 2.57 (s, 3H). Found: [M = H] = 181.1

This Weinreb amide was similarly converted into 3-(dimethylamino)-1-(2-methylpyridin-3-yl)propan-1-one in 75% yield. ^1^H NMR (CDCl_3_) *δ* 8.58 (dd, *J* = 4.9, 1.7 Hz, 1H), 7.90 (dd, *J* = 7.8, 1.7 Hz, 1H), 7.22 (dd, *J* = 7.8, 4.9 Hz, 1H), 3.05 (t, *J* = 7.0 Hz, 2H), 2.71 (t, *J* = 7.0 Hz, 2H), 2.71 (s, 3H), 2.25 (s, 6H). Found: [M+H] = 193.0

##### 4.1.1.9 3-(Dimethylamino)-1-(4-methylpyridin-3-yl)propan-1-one (Class I)

This was prepared similarly from 4-methylnicotinic acid to give *N*-methoxy-*N*,5-dimethylnicotinamide in 95% yield. ^1^H NMR (CDCl_3_) *δ* 8.87 (d, *J* = 1.8 Hz, 1H), 7.95 (dd, *J* = 8.0, 2.2 Hz, 1H), 7.21 (d, *J* = 8.0 Hz, 1H), 3.56 (s, 3H), 3.38 (s, 3H), 2.61 (s, 3H). Found: [M+H] = 181.1

This Weinreb amide was similarly converted into 3-(dimethylamino)-1-(4-methylpyridin-3-yl)propan-1-one in 97% yield. ^1^H NMR (CDCl_3_) *δ* 9.06 (d, *J* = 2.0 Hz, 1H), 8.13 (dd, *J* = 8.1, 2.3 Hz, 1H), 7.27 (d, *J* = 8.1 Hz, 1H), 3.14 (t, *J* = 7.2 Hz, 2H), 2.76 (t, *J* = 7.2 Hz, 2H), 2.63 (s, 3H), 2.29 (s, 6H). Found: [M+H] = 193.0

##### 4.1.1.10 3-(Dimethylamino)-1-(5-methylpyridin-3-yl)propan-1-one (Class J)

This was prepared similarly from 5-methylnicotinic acid to give *N*-methoxy-*N*,5-dimethylnicotinamide in 87% yield. ^1^H NMR (CDCl_3_) *δ* 8.75 (d, *J* = 1.7 Hz, 1H), 8.52 (d, *J* = 1.7 Hz, 1H), 8.03 (m, 1H), 3.57 (s, 3H), 2.85 (s, 3H), 3.17 (s, 3H). Found: [M+H] = 181.

This Weinreb amide was similarly converted into 3-(dimethylamino)-1-(5-methylpyridin-3-yl)propan-1-one in 93% yield. ^1^H NMR (CDCl_3_) *δ* 8.98 (d, *J* = 1.9 Hz, 1H), 8.61 (d, *J* = 1.6 Hz, 1H), 8.03 (m, 1H), 3.15 (t, *J* = 7.0 Hz, 2H), 2.77 (t, *J* = 7.0 Hz, 2H), 2.29 (s, 6H). Found: [M+H] = 193.0.

##### 4.1.1.11 3-(Dimethylamino)-1-(2,6-dimethylpyridin-3-yl)propan-1-one (Class K)

This was prepared similarly from 2,6-dimethylnicotinic acid to give *N*-methoxy-*N*,2,6-trimethylnicotinamide in 66% yield. ^1^H NMR (CDCl_3_) *δ* 7.50 (d, *J* = 7.8 Hz, 1H), 7.02 (d, *J* = 7.8 Hz, 1H), 3.48 (s, 3H), 3.34 (s, 3H), 2.55 (s, 3H), 2.54 (s, 3H). Found: [M+H] = 195.

This Weinreb amide was similarly converted into 3-(dimethylamino)-1-(2,6-dimethylpyridin-3-yl)propan-1-one in 99% yield. ^1^H NMR (CDCl_3_) *δ* 7.84 (d, *J* = 8.0 Hz, 1H), 7.07 (d, *J* = 8.0 Hz, 1H), 3.04 (t, *J* = 7.2 Hz, 2H), 2.70 (t, *J* = 7.2 Hz, 2H), 2.69 (s, 3H), 2.57 (s, 3H), 2.26 (s, 6H). Found: [M+H] = 207.0.

##### 4.1.1.12 4,1.1.12. 3-(Dimethylamino)-1-(5,6-dimethylpyridin-3-yl)propan-1-one (Class L)

This was prepared similarly from 5,6-dimethylnicotinic acid to give *N*-methoxy-*N*,5,6-trimethylnicotinamide in 92% yield. ^1^H NMR (CDCl_3_) *δ* 8.69 (d, *J* = 1.8 Hz, 1H), 7.76 (d, *J* = 1.6 Hz, 1H), 3.57 (s, 3H), 3.37 (s, 3H), 2.55 (s, 3H), 2.32 (s, 3H). Found: [M+H] = 195.1.

This Weinreb amide was similarly converted into 3-(dimethylamino)-1-(5,6-dimethylpyridin-3-yl)propan-1-one in 98% yield. ^1^H NMR (CDCl_3_) *δ* 8.89 (d, *J* = 2.1 Hz, 1H), 7.95 (d, *J* = 1.6 Hz, 1H), 3.13 (t, *J* = 7.3 Hz, 2H), 2.76 (t, *J* = 7.3 Hz, 2H), 2.57 (s, 3H), 2.35 (s, 3H), 2.29 (s, 6H). Found: [M+H] = 207.0.

##### 4.1.1.13 3-(Dimethylamino)-1-(4,6-dimethylpyridin-3-yl)propan-1-one (Class M)

This was prepared similarly from 4,6-dimethylnicotinic acid to give *N*-methoxy-*N*,4,6-trimethylnicotinamide in 93% yield. ^1^H NMR (CDCl_3_) *δ* 8.40 (s, 1H), 7.03 (s, 1H), 3.50 (s, 3H), 3.48 (s, 3H), 2.54 (s, 3H), 2.32 (s, 3H). Found: [M+H] = 195.1.

This Weinreb amide was similarly converted into 3-(dimethylamino)-1-(4,6-dimethylpyridin-3-yl)propan-1-one in 99% yield. ^1^H NMR (CDCl_3_) *δ* 8.82 (s, 1H), 7.05 (s, 1H), 3.09 (t, *J* = 7.2 Hz, 2H), 2.72 (t, *J* = 7.2 Hz, 2H), 2.55 (s, 3H), 2.50 (s, 3H), 2.26 (s, 6H). Found: [M+H] = 207.1.

##### 4.1.1.14 3-(Dimethylamino)-1-(2-methoxypyridin-3-yl)propan-1-one (Class N)

This was prepared similarly from 2-methoxynicotinic acid to give *N*,2-dimethoxy-*N*-methylnicotinamide in 78% yield. ^1^H NMR (CDCl_3_) *δ* 8.22 (dd, *J* = 5.0, 1.8 Hz, 1H), 7.60 (bd, *J* = 6.3 Hz, 1H), 6.93 (dd, *J* = 7.2, 5.0 Hz, 1H), 3.99 (s, 3H), 3.54 (bs, 3H), 3.33 (bs, 3H). Found: [M+H] = 197.0.

The Weinreb amide was similarly converted to 3-(dimethylamino)-1-(2-methoxypyridin-3-yl)propan-1-one in 95% yield. ^1^H NMR (CDCl_3_) *δ* 8.30 (dd, *J* = 4.8, 2.0 Hz, 1H), 8.08 (dd, *J* = 7.6, 2.0 Hz, 1H), 6.98 (dd, *J* = 7.5, 4.8 Hz, 1H), 4.05 (s, 3H), 3.21 (t, *J* = 7.1 Hz, 2H), 2.71 (t, *J* = 7.1 Hz, 2H), 2.27 (s, 6H). Found: [M+H] = 209.1.

##### 4.1.1.15 3-(Dimethylamino)-1-(6-methoxypyridin-3-yl)propan-1-one (Class O)

This was prepared similarly from 6-methoxynicotinic acid to give *N*,6-dimethoxy-*N*-methylnicotinamide in 83% yield. ^1^H NMR (CDCl_3_) *δ* 8.65 (dd, *J* = 2.4, 0.5 Hz, 1H), 8.00 (dd, *J* = 8.7, 2.4 Hz, 1H), 6.76 (dd, *J* = 8.7, 0.7 Hz, 1H), 3.99 (s, 3H), 3.58 (s, 3H), 3.38 (s, 3H). Found: [M+H] = 197.0.

This Weinreb amide was similarly converted into 3-(dimethylamino)-1-(6-methoxypyridin-3-yl)propan-1-one in 99% yield. ^1^H NMR (CDCl_3_) *δ* 8.81 (dd, *J* = 2.3, 0.4 Hz, 1H), 8.15 (dd, *J* = 8.7, 2.4 Hz, 1H), 6.79 (dd, *J* = 8.7, 0.6 Hz, 1H), 4.00 (s, 3H), 3.09 (t, *J* = 7.3 Hz, 2H), 2.75 (t, *J* = 7.3 Hz, 2H), 2.29 (s, 6H). Found: [M+H] = 209.0.

##### 4.1.1.16 1-(2,5-Dimethoxypyridin-3-yl)-3-(dimethylamino)propan-1-one (Class P)

This was prepared similarly from 2,5-dimethoxynicotinic acid to give *N*,2,5-trimethoxy-*N*-methylnicotinamide in 61% yield. ^1^H NMR (CDCl_3_) *δ* 7.87 (br s, 1H), 7.24 (br s, 1H), 3.94 (s, 3H), 8.82 (s, 3H), 3.56 (br s, 3H), 3.32 (br s, 3H). Found: [M+H] = 227.0.

This Weinreb amide was similarly converted into 1-(2,5-dimethoxypyridin-3-yl)-3-(dimethylamino)propan-1-one in 90% yield. ^1^H NMR (CDCl_3_) *δ* 8.00 (d, *J* = 3.2 Hz, 1H), 7.69 (d, *J* = 3.2 Hz, 1H), 4.01 (s, 3H), 3.83 (s, 3H), 3.22 (t, *J* = 7.1 Hz, 2H), 2.70 (t, *J* = 7.1 Hz, 2H), 2.27 (s, 6H). Found: [M+H] = 239.2.

##### 4.1.1.17 1-(5,6-Dimethoxypyridin-3-yl)-3-(dimethylamino)propan-1-one (Class Q)

This was prepared similarly from 5,6-dimethoxynicotinic acid to give *N*,5,6-trimethoxy-*N*-methylnicotinamide in 85% yield. ^1^H NMR (CDCl_3_) *δ* 8.26 (d, *J* = 1.9 Hz, 1H), 7.49 (d, *J* = 1.9 Hz, 1H), 4.11 (s, 3H), 3.91 (s, 3H), 3.61 (s, 3H), 3.39 (s, 3H). Found: [M+H] = 227.1.

The Weinreb amide was similarly converted to 1-(5,6-dimethoxypyridin-3-yl)-3-(dimethylamino)prpoan-1-one in 32% yield. ^1^H NMR (CDCl_3_) *δ* 8.40 (d, *J* = 1.9 Hz, 1H), 7.61 (d, *J* = 1.9 Hz, 1H), 4.09 (s, 3H), 3.92 (s, 3H), 3.11 (t, *J* = 7.1 Hz, 2H), 2.77 (t, *J* = 7.1 Hz, 2H), 2.30 (s, 6H). Found: [M+H] = 239.1.

##### 4.1.1.18 3-(Dimethylamino)-1-(2,4,5-trimethoxypyridin-3-yl)propan-1-one (Class R)

This was prepared similarly from 2,4,5-trimethoxynicotinic acid to give *N*,2,4,5-tetramethoxy-*N*-methylnicotinamide in 94% yield. ^1^H NMR (CDCl_3_) *δ* 7.20 (s, 1H), 4.03 (s, 3H), 3.94 (s, 3H), 3.83 (s, 3H), 3.64 (br s, 3H), 3.30 (s, 3H). Found [M+H] = 257.1.

This Weinreb amide was similarly converted into 3-(dimethylamino)-1-(2,4,5-trimethoxypyridin-3-yl)propan-1-one in 92% yield. . ^1^H NMR (CDCl_3_) *δ* 7.71 (s, 1H), 4.07 (s, 3H), 4.02 (s, 3H), 3.86 (s, 3H), 3.19 (t, *J* = 7.1 Hz, 2H), 2.70 (t, *J* = 7.1 Hz, 2H), 2.29 (s, 6H). Found: [M+H] = 269.2.

##### 4.1.1.19 3-(Dimethylamino)-1-(3-methylpyridin-4-yl)propan-1-one (Class S)

This was prepared similarly from 3-methylisonicotinic acid to give *N*-methoxy-*N*,3-dimethylisonicotinamide in 90% yield. ^1^H NMR (CDCl_3_) *δ* 8.51 (s, 1H), 8.49 (d, *J* = 4.9 Hz, 1H), 7.18 (d, *J* = 4.9 Hz, 1H)), 3.38 (s, 3H), 3.37 (s, 3H), 2.32 (s, 3H). Found: [M+H] = 181.1.

This Weinreb amide was similarly converted into 3-(dimethylamino)-1-(3-methylpyridin-4-yl)propan-1-one in 31% yield. ^1^H NMR (CDCl_3_) *δ* 8.57 (dd, *J* = 5.0, 0.4 Hz, 1H), 8.55 (s, 1H), 7.37 (d, *J* = 5.0 Hz, 1H), 3.03 (t, *J* = 7.1 Hz, 2H), 2.69 (t, *J* = 7.1 Hz, 2H), 2.43 (s, 3H), 2.24 (s, 6H). Found: [M+H] = 193.0.

##### 4.1.1.20 3-(Dimethylamino)-1-(2-methylpyridin-4-yl)propan-1-one (Class T)

This was prepared similarly from 2-methylisonicotinic acid to give *N*-methoxy-*N*,2-dimethylisonicotinamide in 79% yield. ^1^H NMR (CDCl_3_) *δ* 8.58 (dd, *J* = 5.0, 0.5 Hz, 1H), 7.37 (s, 1H), 7.30 (d, *J* = 5.0 Hz, 1H), 3.55 (s, 3H), 3.36 (s, 3H), 2.61 (s, 3H). Found: [M+H] = 181.1.

This Weinreb amide was similarly converted into 3-(dimethylamino)-1-(2-methylpyridin-4-yl)propan-1-one in 99% yield. ^1^H NMR (CDCl_3_) *δ* 8.68 (dd, *J* = 5.1, 0.4 Hz, 1H), 7.59 (s, 1H), 7.52 (dd, *J* = 5.1, 1.0 Hz, 1H), 3.12 (t, *J* = 7.2 Hz, 2H), 2.75 (t, *J* = 7.2 Hz, 2H), 2.64 (s, 3H), 2.28 (s, 6H). Found: [M+H] = 193.1.

##### 4.1.1.21 3-(Dimethylamino)-1-(2,6-dimethylpyridin-4-yl)propan-1-one (Class U)

This was prepared similarly from 2,6-dimethylisonicotinic acid to give *N*-methoxy-*N*,2,6-trimethylisonicotinamide in 49% yield. ^1^H NMR (CDCl_3_) *δ* 7.15 (s, 2H), 3.56 (s, 3H), 3.35 (s, 3H), 2.57 (s, 6H). Found: [M+H] = 195.0.

This Weinreb amide was similarly converted into 3-(dimethylamino)-1-(2,6-dimethylpyridin-4-yl)propan-1-one in 99% yield. ^1^H NMR (CDCl_3_) *δ* 7.38 (s, 2H), 3.10 (t, *J* = 7.2 Hz, 2H), 2.74 (t, *J* = 7.2 Hz, 2H), 2.61 (s, 6H), 2.28 (s, 6H). Found: [M+H] = 207.1.

##### 4.1.1.22 1-(2,6-Diethoxypyridin-4-yl)-3-(dimethylamino)propan-1-one (Class V)

This was prepared similarly from 2,6-diethoxyisonicotinic acid to give 2,6-diethoxy-*N*-methoxy-*N*-methylisonicotinamide in 69% yield. ^1^H NMR (CDCl_3_) *δ* 6.43 (s, 2H), 4.33 (q, *J* = 7.1 Hz, 2H), 3.59 (br s, 3H), 3.32 (s, 3H), 1.39 (t, *J* = 7.1 Hz, 3H). Found: [M+H] = 255.1.

This Weinreb amide was similarly converted into 3-(dimethylamino)-1-(2,6-dimethylpyridin-4-yl)propan-1-one in 96% yield. ^1^H NMR (CDCl_3_) *δ* 6.71 (s, 2H), 4.34 (q, *J* = 7.1 Hz, 2H), 3.05 (t, *J* = 7.0 Hz, 2H), 2.72 (t, *J* = 7.0 Hz, 2H), 2.26 (s, 6H), 1.40 (t, *J* = 7.0 Hz, 3H). Found: [M+H] = 267.0.

##### 4.1.1.23 1-(2,5-Dimethoxypyridin-4-yl)-3-(dimethylamino)propan-1-one (Class W)

This was prepared similarly from 2,5-dimethoxyisonicotinic acid to give *N*,2,5-trimethoxy-*N*-methylisonicotinamide in 41% yield. ^1^H NMR (CDCl_3_) *δ* 7.82 (s, 1H), 6.60 (br s, 1H), 3.90 (s, 3H), 3.89 (s, 3H), 3.51 (br s, 1H), 3.35 (br s, 3H). Found: [M+H] = 227.1.

This Weinreb amide was similarly converted into 1-(2,5-dimethoxypyridin-4-yl)-3-(dimethylamino)propan-1-one in 67% yield. ^1^H NMR (CDCl_3_) *δ* 7.89 (s, 1H), 6.86 (d, *J* = 0.4 Hz, 1H), 3.91 (s, 3H), 3.90 (s, 3H), 3.10 (t, *J* = 7.1 Hz, 2H), 2.67 (t, *J* = 7.1 Hz, 2H), 2.24 (s, 6H). Found: [M+H] = 239.2.

##### 4.1.1.24 3-(Dimethylamino)-1-(2-methoxy-6-propoxypyridin-4-yl)propan-1-one (Class X).

A solution of methyl 2-hydroxy-6-methoxyisonicotinate (6.00 g, 32.8 mmol) in DMF (100 mL, anhydrous) was treated with K_2_CO_3_ (6.80 g, 49.2 mmol) and then 1-iodopropane (4.8 mL, 49.2 mmol). The mixture was stirred at 20 °C for 48 h, partitioned between EtOAc and water and the aqueous layer was extracted with EtOAc. The organic fractions were washed with water, dried and evaporated. Column chromatography (DCM) gave methyl 2-methoxy-6-propoxyisonicotinate (6.59 g, 89%). ^1^H NMR (CDCl_3_) *δ* 6.85 (d, *J* = 1.0 Hz, 1H), 6.83 (d, *J* = 1.0 Hz, 1H), 4.24 (t, *J* = 6.7 Hz, 2H), 3.93 (s, 3H) , 3.91 (s, 3H), 1.80 (qt, *J* = 7.4, 6.7 Hz, 2H), 1.02 (t, *J* = 7.4 Hz, 3H). Found: [M+H] = 226.1.

A solution of LiOH (2.04 g, 85.2 mmol) in water (60 mL) was added to a solution of methyl 2-methoxy-6-propoxyisonicotinate (6.40 g, 28.4 mmol) in THF (60 mL) and MeOH (60 mL), the solution was stirred at 20 °C for 18 h and then evaporated. The residue was dissolved in water (200 mL) and acidified to pH 3 with 2 M HCl. The precipitate was filtered and dried to give 2-methoxy-6-propoxyisonicotinic acid (5.39 g, 90%). ^1^H NMR (DMSO‑*d*_6_) *δ* 13.53 (bs, 1H), 6.73 (d, *J* = 1.0 Hz, 1H), 6.72 (d, *J* = 1.0 Hz, 1H), 4.23 (t, *J* = 6.6 Hz, 2H), 3.87 (s, 3H), 1.73 (qt, *J* = 7.4, 6.6 Hz, 2H), 0.96 (t, *J* = 7.4 Hz, 3H). Found: [M+H] = 212.1.

Oxalyl chloride (2.76 mL, 32.6 mmol) was added to 2-methoxy-6-propoxyisonicotinic acid (5.74 g, 27.2 mmol) in DCM (100 mL, anhydrous) and DMF (0.4 mL, 5.2 mmol) at 20 °C. The mixture was stirred at 20 °C for 1 h to give a colourless solution which was cooled to 0 °C. *N*,*O*-Dimethylhydroxylamine hydrochloride (3.18 g, 32.6 mmol) and pyridine (6.6 mL, 81.6 mmol) were added sequentially and the mixture was stirred at 20 °C for 18 h, then partitioned between DCM and water. Column chromatography on alumina with DCM gave *N*,2-dimethoxy-*N*-methyl-6-propoxyisonicotinamide (6.29 g, 91%). ^1^H NMR (CDCl_3_) *δ* 6.42 (s, 1H), 6.41 (s, 1H), 5.24 (sp, *J* = 6.2 Hz, 1H), 3.90 (s, 3H), 3.59 (bs, 3H), 3.32 (s, 3H), 1.35 (d, *J* = 6.2 Hz, 6H). Found: [M+H] = 255.1.

Vinylmagnesium bromide (43 mL, 1 M in THF, 43 mmol) was added to a solution of *N*,2-dimethoxy-*N*-methyl-6-propoxyisonicotinamide (5.78 g, 21.7 mmol) in THF (100 mL, dist. Na) at 0 °C, the brown solution was stirred at 0 °C for 1 h and then dimethylamine (43 mL, 2 M in THF, 86 mmol) and water (40 mL) were added. The solution was stirred at 20 °C for 1 h then partitioned between EtOAc and water. The solution was dried and evaporated to give 3-(dimethylamino)-1-(2-methoxy-6-propoxypyridin-4-yl)propan-1-one (5.75 g, 100%). ^1^H NMR (CDCl_3_) *δ* 6.73 (d, *J* = 1.1 Hz, 1H), 6.72 (d, *J* = 1.1 Hz, 1H), 4.26 (t, *J* = 6.7 Hz, 2H), 3.93 (s, 3H), 3.06 (t, *J* = 7.4 Hz, 2H), 2.72 (t, *J* = 7.4 Hz, 2H), 2.27 (s, 6H), 1.80 (qt, *J* = 7.4, 6.7 Hz, 2H), 1.03 (t, *J* = 7.4 Hz, 3H). Found: [M+H] = 267.2.

##### 4.1.1.25 3-(Dimethylamino)-1-(2-ethoxy-6-isopropoxypyridin-4-yl)propan-1-one (Class X)

A suspension of 2,6-dihydroxyisonicotinic acid (40.00 g, 258 mmol) in EtOH (300 mL) was treated dropwise with H_2_SO_4_ (40 mL, 18.4 M, 752 mmol). The solution was refluxed for 72 h then evaporated; the residue was treated with sat. aq. NaHCO_3_ to pH 8 and then extracted with EtOAc (3 × 500 mL). The organic extracts were washed with sat. aq. NaHCO_3_, brine, then dried and evaporated to give ethyl 2-ethoxy-6-hydroxyisonicotinate (10.86 g, 20%). ^1^H NMR (DMSO‑*d*_6_) *δ* 11.15 (bs, 1H), 6.59 (d, *J* = 1.0 Hz, 1H), 6.57 (bs, 1H), 4.29 (q, *J* = 7.1 Hz, 2H), 4.25 (q, *J* = 7.1 Hz, 2H), 1.298 (t, *J* = 7.1 Hz, 3H), 1.296 (t, *J* = 7.1 Hz, 3H). Found: [M+H] = 212.2.

A solution of ethyl 2-ethoxy-6-hydroxyisonicotinate (10.82 g, 51.2 mmol) in DMF (125 mL, anhydrous) was treated with K_2_CO_3_ (8.65 g, 62.5 mmol) and then 2-iodopropane (6.4 mL, 64 mmol). The mixture was stirred at 20 °C. for 48 h, K_2_CO_3_ (8.65 g, 62.5 mmol) and 2-iodopropane (6.4 mL, 64 mmol) were added and the mixture was stirred for a further 24 h, partitioned between EtOAc and water and the aqueous layer was extracted with EtOAc. The organic fractions were washed with water, dried and evaporated. Chromatography (DCM) gave ethyl 2-ethoxy-6-isopropoxyisonicotinate (11.56 g, 89%). ^1^H NMR (DMSO‑*d*_6_) *δ* 6.81 (s, 2H), 5.23 (sp, *J* = 6.2 Hz, 1H), 4.20–4.38 (m, 4H), 1.35–1.42 (m, 12H). Found: [M+H] = 254.1.

A solution of LiOH (3.25 g, 136 mmol) in water (60 mL) was added to a solution of ethyl 2-ethoxy-6-isopropoxyisonicotinate (11.42 g, 45.1 mmol) in THF (60 mL) and MeOH (60 mL), the solution was stirred at 20 °C for 18 h and then evaporated. The residue was dissolved in water (150 mL) and acidified to pH 3 with 2 M HCl. The precipitate was filtered and dried to give 2-ethoxy-6-isopropoxyisonicotinic acid (10.03 g, 99%). ^1^H NMR (DMSO‑*d*_6_) *δ* 13.48 (bs, 1H), 6.66 (d, *J* = 0.9 Hz, 1H), 6.64 (d, *J* = 0.9 Hz, 1H), 5.17 (sp, *J* = 6.2 Hz, 1H), 4.29 (q, *J* = 7.0 Hz, 2H), 1.32 (t, *J* = 7.0 Hz, 3H), 1.30 (d, *J* = 6.2 Hz, 6H). Found: [M+H] = 226.1.

Oxalyl chloride (3.13 mL, 37 mmol) was added to 2-ethoxy-6-isopropoxyisonicotinic acid (6.95 g, 30.8 mmol) in DCM (100 mL, anhydrous) and DMF (5.2 mmol) at 20 °C. The mixture was stirred at 20 °C for 1 h to give a colourless solution which was cooled to 0 °C. *N*,*O*-Dimethylhydroxylamine hydrochloride (3.61 g, 37.0 mmol) and pyridine (7.5 mL, 92.7 mmol) were added sequentially and the mixture was stirred at 20 °C for 18 h, then partitioned between EtOAc and water. The organic fractions were washed with water, dried and evaporated. Column chromatography with 95:5 DCM:EtOAc gave 2-ethoxy-6-isopropoxy-*N*-methoxy-*N*-methylisonicotinamide (7.98 g, 97%). ^1^H NMR (CDCl_3_) *δ* 6.40 (s, 1H), 6.39 (s, 1H), 5.22 (sp, *J* = 6.2 Hz, 1H), 4.33 (q, *J* = 7.1 Hz, 2H), 3.60 (bs, 3H), 3.31 (s, 3H), 1.39 (t, *J* = 6.2 Hz, 3H), 1.34 (d, *J* = 7.1 Hz, 6H). Found: [M+H] = 269.2.

Vinylmagnesium bromide (40 mL, 1 M in THF, 40 mmol) was added to a solution of 2-ethoxy-6-isopropoxy-*N*-methoxy-*N*-methylisonicotinamide (5.33 g, 19.9 mmol) in THF (100 mL, dist. Na) at 0 °C, the brown solution was stirred at 0 °C for 1 h and then dimethylamine (40 mL, 2 M in THF, 80 mmol) and water (40 mL) were added. The solution was stirred at 20 °C. for 1 h then partitioned between EtOAc and water. The solution was dried and evaporated, to give 3-(dimethylamino)-1-(2-ethoxy-6-isopropoxypyridin-4-yl)propan-1-one (5.57 g, 100%) as a brown oil. ^1^H NMR (CDCl_3_) *δ* 6.68 (d, *J* = 1.0 Hz, 1H), 6.67 (d, *J* = 1.0 Hz, 1H), 5.22 (sp, *J* = 6.2 Hz, 1H), 4.33 (q, *J* = 7.0 Hz, 2H), 3.05 (t, *J* = 7.5 Hz, 2H), 2.72 (t, *J* = 7.5 Hz, 2H), 2.26 (s, 6H), 1.40 (t, *J* = 6.2 Hz, 3H), 1.36 (d, *J* = 7.0 Hz, 6H). Found: [M+H] = 281.7.

#### 4.1.2 Scheme 1: Preparation of the 6-bromo compounds of Table 1 example of 1-(7-bromo-3-methoxynaphthalen-2-yl)-1-(2,3-dimethoxyphenyl)-4-(dimethylamino)-2-(2,6-dimethylpyridin-4-yl)butan-2-ol (**56**)

A solution of dry diisopropylamine (1.30 mL, 9.28 mmol) in dry THF (10 mL) was cooled to −40 °C under an atmosphere of dry nitrogen. *N*-Butyllithium (4.65 mL of a 2.0 N solution in cyclohexane, 9.28 mmol) was added dropwise, then stirring was continued for a further 15 min. The solution was cooled to −70 to −78 °C and a solution of 6-bromo-3-(2,3-dimethoxybenzyl)-2-methoxyquinoline^6^ (3.00 g, 7.73 mmol) in dry THF (6 mL) was added dropwise. The resulting purple solution was stirred at this temperature for 90 min. A solution of 3-(dimethylamino)-1-(2,6-dimethylpyridin-4-yl)propan-1-one (class U Mannich base) (1.75 g, 8.50 mmol) in THF (6 mL) was then added dropwise and the mixture was stirred at this temperature for 5 h. Glacial acetic acid (0.70 mL) was added in one portion and the mixture was allowed to warm to room temperature. Water was added and the mixture was extracted with EtOAc. The extract was washed with water and dried over sodium sulfate. Removal of the solvent under reduced pressure left an oil, which was chromatographed on silica. Column chromatography with DCM gave fore fractions, followed by isomer A of **56** (1.90 g, 41%).

Isomer A, white solid. ^1^H NMR (CDCl_3_, 400 MHz) *δ* 8.21 (s, 1H), 7.83 (d, *J* = 2.2 Hz, 1H), 7.71–7.66 (m, 2H), 7.60 (dd, *J* = 8.9, 2.2 Hz, 1H), 7.20–7.13 (m, 3H), 6.83 (t, *J* = 8.1 Hz, 1H), 6.59 (dd, *J* = 8.2, 1.4 Hz, 1H), 5.60 (s, 1H), 4.23 (s, 3H), 3.68 (s, 3H), 3.47 (s, 3H), 2.47 (s, 6H), 2.28–2.20 (m, 1H), 2.00–1.96 (m, 1H), 1.99 (s, 6H), 1.85–1.80 (m, 2H). Found: [M+H] = 594.6.

Elution with DCM:MeOH (92:8) gave isomer B (1.01 g, 22%).

Isomer B, white solid. ^1^H NMR (CDCl_3_, 400 MHz) *δ* 8.65 (s, 1H), 7.81 (d, *J* = 2.1 Hz, 1H), 7.57 (dd, *J* = 8.9, 2.0 Hz, 1H), 7.62 (d, *J* = 8.9 Hz, 1H), 7.28–7.21 (m, 4H), 7.05 (t, *J* = 8.0 Hz, 1H), 6.87 (d, *J* = 8.6 Hz, 1H), 4.95 (s, 1H), 3.99 (s, 3H), 3.90 (s, 3H), 3.90 (s, 3H), 2.23–2.18 (m, 2H), 2.43 (s, 6H), 2.03 (s, 6H), 1.95–1.80 (m, 2H). Found: [M+H] = 594.6.

Each coupled product was resolved into its four optical isomers using preparative chiral HPLC at BioDuro LLC (Beijing).

The other 6-bromo analogues of Table 1 were prepared similarly:

#### 4.1.3 Scheme 1: Preparation of the 6-cyano compounds of Table 1 7-(1-(2,3-Dimethoxyphenyl)-4-(dimethylamino)-2-(2,6-dimethylpyridin-4-yl)-2-hydroxybutyl)-6-methoxy-2-naphthonitrile (**57**)

A solution of **56** (1.62 g, 2.73 mmol) in DMF (10 mL, anhydrous) was purged with nitrogen and heated to 55 °C for 10 min. Tri(*o*-tolyl)phosphine (0.125 g, 0.41 mmol), zinc dust (0.018 g, 0.273 mmol) and tris(dibenzylideneacetone)dipalladium(0) (0.188 g, 0.205 mmol) were then added, and the reaction was again purged with nitrogen and heated for another 10 min at 55 °C. Zinc cyanide (0.177 g, 1.50 mmol) was then added and the reaction mixture was heated to 65 °C for 4 h. The reaction was diluted with water and extracted with EtOAc three times. The organic layer was washed with brine three times, dried and evaporated. Column chromatography with DCM followed afforded isomer A of **57** (0.65 g, 44%) followed by isomer B of **57** (0.46 g, 31%) as white solids.

Isomer A, white solid. ^1^H NMR (CDCl_3_, 400 MHz) *δ* 8.33 (s, 1H), 8.16 (s, 1H), 8.06 (d, *J* = 1.8 Hz, 1H), 7.85 (d, *J* = 8.6 Hz, 1H), 7.73–6.98 (m, 2H), 7.21–7.11 (m, 2H), 6.84 (t, *J* = 8.1 Hz, 1H), 6.60 (dd, *J* = 8.2, 1.4 Hz, 1H), 5.60 (s, 1H), 4.28 (s, 3H), 3.68 (s, 3H), 3.49 (s, 3H), 2.47 (s, 6H), 2.21–2.13 (m, 1H), 2.02–1.96 (m, 1H), 1.95 (s, 6H), 1.85–1.72 (m, 2H). Found: [M+H] = 542.4.

Isomer B, white solid. ^1^H NMR (CDCl_3_, 400 MHz) *δ* 8.79 (s, 1H), 8.21 (s, 1H), 8.03 (d, *J* = 1.7 Hz, 1H), 7.68 (d, *J* = 8.6 Hz, 1H), 7.62 (dd, *J* = 8.6, 1.8 Hz, 1H), 7.37 (dd, *J* = 8.0, 1.4 Hz, 1H), 7.13–7.06 (m, 2H), 7.00 (t, *J* = 8.0 Hz, 1H), 6.82 (dd, *J* = 8.0, 1.4 Hz, 1H), 5.54 (s, 1H), 4.01 (s, 3H), 3.90 (s, 3H), 3.90 (s, 3H), 2.43 (s, 6H), 2.20–2.12 (m, 1H), 2.02 (s, 6H), 2.01–1.93 (m, 2H), 1.74–1.68 (m, 1H). Found: [M+H] = 542.4.

In each case, the coupled 6-cyano product was then resolved into its four optical isomers using preparative chiral HPLC at BioDuro LLC (Beijing).

The other 6-cyano analogues of Table 1 were prepared similarly:

## Appendix A Supplementary data

Supplementary Data 1 MOL files for the compounds of Table 2.

### MOL files

The following ZIP file contains the MOL files of the most important compounds referred to in this article.

MOL files ZIP file containing the MOL file of the most important compounds in this article
