# Supplementary material for: Structure-activity relationships for unit C pyridyl analogues of the tuberculosis drug bedaquiline
Source: Bioorg Med Chem. 2019 Apr 1;27(7):1283–91. doi: 10.1016/j.bmc.2019.02.025 (PMC6467542; doi:10.1016/j.bmc.2019.02.025)
Supplement: Supplementary Data 1 — MOL files for the compounds of Table 2. [file mmc1.docx]

Supplementary Material for

**Structure-activity relationships for unit C pyridyl analogues of the tuberculosis drug bedaquiline**

Adrian Blaser^a^, Hamish S. Sutherland^a^, Amy S.T. Tong^a^, Peter J. Choi^a^, Scott G. Franzblau^c^, Manisha U. Lotlikar^d^, Christopher B. Cooper^d^, Anna M. Upton^d^, William A. Denny^a,b.*^, Brian D. Palmer^a,b^

**Index**

| **Section** | **page** |
| --- | --- |
| Scheme 1; General synthesis of bedaquiline analogues | 1 |
| Scheme 2: Synthesis of Mannich bases | 2 |
| Table 1: Details for new Mannich bases | 2 |
| Syntheses of new Mannich bases (IUPAC numbering) | 3-11 |
| Representative synthesis of the 6-bromo analogues of Table 2 | 11 |
| Representative synthesis of the 6-cyano analogues of Table 2 | 12 |
| References | 13 |

**Scheme 1: General synthesis of bedaquiline analogues**

Syntheses of all the required A/B units have been published previously (refs S1-S4).

**Scheme 2: Synthesis of pyridyl 3-(dimethylamino)propan-1-one Mannich bases**

Reagents and conditions: (i) (COCl_2_, cat. DMF, DCM, then MeNH(OMe).HCl, pyridine; (ii) vinylMgBr, Me_2_NH, THF.

**Supplementary Table 1: Details for new Mannich bases**

| **class** | **N** | **X**^a^ | **% yields I/II/III** |
| --- | --- | --- | --- |
| A | 2- | 3-Me | 95/97 |
| B | 2- | 5-Me | 31/99 |
| C | 2- | 6-Me | 46/99 |
| D | 2- | 3,5-diMe | 88/98 |
| E | 2- | 4,6-diMe | 23/99 |
| F | 2- | 3-OMe | 92/95 |
| G | 2- | 5-OMe | 72/99 |
| H | 3- | 2-Me | 83/75 |
| I | 3- | 4-Me | 95/97 |
| J | 3- | 5-Me | 87/93 |
| K | 3- | 2,4-diMe | 66/99 |
| L | 3- | 4,5-diMe | 92/98 |
| M | 3- | 4,6-diMe | 93/99 |
| N | 3- | 2-OMe | 78/99 |
| O | 3- | 4-OMe | 83/99 |
| P | 3- | 2,5-diOMe | 61/90 |
| Q | 3- | 4,5-diOMe | 85/83 |
| R | 3- | 2,4,5-triOMe | 94/92 |
| S | 4- | 2-Me | 90/31 |
| T | 4- | 3-Me | 79/99 |
| U | 4- | 3,5-diMe | 62/99 |
| V | 4- | 3,5-diEt | 82/99 |
| W | 4- | 2,5-diOMe | 41/67 |

Footnotes for Supplementary Table 1: ^a^For clarity, substituent numbering is consistent with that in Table 1; is not necessarily IUPAC.

**Syntheses of new Mannich bases (IUPAC numbering used)**

**Class A: 3-(Dimethylamino)-1-(6-methylpyridin-2-yl)propan-1-one**

Oxalyl chloride (1.85mL, 21.88 mmol) was added to a suspension of 6-methylpicolinic acid (2.50g, 18.23 mmol) in DCM (75 mL, anhydrous) and DMF (1.3 mmol) at r.t.. The mixture was stirred at r.t. for 1 h to give a colorless solution which was cooled to 0 °C. *N,O*-Dimethylhydroxylamine hydrochloride (1.95 g, 20.1 mmol) and pyridine (3.25 mL, 40.11 mmol) were added sequentially and the mixture was stirred at r.t. for 18 h, then partitioned between EtOAc and sat. aq. NaHCO_3_. Column chromatography with hexanes:EtOAc 1:1 gave N-methoxy-N,6-dimethylpicolinamide as an oil (2.68 g, 82%). ^1^H NMR (CDCl_3_) δ 7.66 (t, *J* = 7.8 Hz, 1H), 7.44 (s, 1H), 7.21 (dd, *J* = 7.6, 0.4 Hz), 3.76 (s, 3H), 3.39 (s, 3H), 2.59 (s, 3H). Found: [M+H]=181.

Vinylmagnesium bromide solution in THF (1N, 45 mL, 44.6 mmol) was added to a solution of N-methoxy-N,6-dimethylpicolinamide (2.68 g, 14.87 mmol) in THF (130 mL, dist. Na) at 0 °C, the brown solution was warmed to r.t. for 1 h then dimethylamine in THF (2N, 45 mL, 89.2 mmol) and water (23 mL) were added. The solution was stirred at r.t. for 1 h, then partitioned between EtOAc and water. The solution was dried and evaporated to give 3-(dimethylamino)-1-(6-methylpyridin-2-yl)propan-1-one as a yellow oil (2.78 g, 97%). ^1^H NMR (CDCl_3_) δ 7.83 (dd, *J* = 7.7, 0.4 Hz, 1H), 7.69 (t, *J* = 7.7 Hz, 1H), 7.31 (dd, *J* = 7.7, 0.4 Hz, 1H), 3.40 (t, *J* = 7.2 Hz, 2H), 2.77 (t, *J* = 7.2 Hz, 2H), 2.61 (s, 3H), 2.29 (s, 6H). Found: [M+H]=193.

**Class B: 3-(Dimethylamino)-1-(4-methylpyridin-2-yl)propan-1-one**

Similarly was prepared, starting from 4-methylpicolinic acid, N-methoxy-N,4-dimethylpicolinamide in 31% yield. ^1^H NMR (CDCl_3_) δ 8.46 (d, *J* = 5.0 Hz, 1H), 7.49 (br, 1H), 7.17 (dd, *J* = 5.0, 0.8 Hz, 1H), 3.77 (br s, 3H), 3.40 (br s, 3H), 2.40 (s, 3H). Found: [M+H]=181.

This Weinreb amide was similarly converted into 3-(dimethylamino)-1-(4-methylpyridin-2-yl)propan-1-one in 99 % yield. ^1^H NMR (CDCl_3_) δ 8.53 (d, *J* = 4.9 Hz, 1H), 7.86 (m, 1H), 7.28 (m, 1H), 3.39 (t, *J* = 7.2 Hz, 2H), 2.77 (t, *J* = 7.2 Hz, 2H), 2.42 (s, 3H), 2.84 (s, 6H). Found: [M+H]=193.

**Class C: 3-(Dimethylamino)-1-(3-methylpyridin-2-yl)propan-1-one**

Similarly was prepared, starting from 3-methylpicolinic acid, N-methoxy-N,3-dimethylpicolinamide in 46% yield. ^1^H NMR (CDCl_3_) δ 8.44 (dd, *J* = 4.7, 0.8 Hz, 1H), 7.56 (d, *J* = 7.6 Hz, 1H), 7.25 (dd, *J* = 7.7, 4.8 Hz, 1H), 3.56 (s, 3H), 3.40 (s, 3H), 2.35 (s, 3H). Found: [M+H]=181.

This Weinreb amide was similarly converted into 3-(dimethylamino)-1-(3-methylpyridin-2-yl)propan-1-one in 99% yield. ^1^H NMR (CDCl_3_) δ 8.50 (dd, *J* = 4.6, 1.1 Hz, 1H), 7.57 (dq, *J* = 7.8, 0.7 Hz, 1H), 7.31 (dd, *J* = 7.7, 4.6 Hz, 1H), 3.36 (t, *J* = 7.2 Hz, 2H), 2.73 (t, *J* = 7.2 Hz, 2H), 2.56 (s, 3H), 2.27 (s, 6H). Found: [M+H]=193.

**Class D: 3-(Dimethylamino)-1-(4,6-dimethylpyridin-2-yl)propan-1-one**

Similarly was prepared, from 4,6-dimethylpicolinic acid, N-methoxy-N,4,6-trimethylpicolinamide in 88% yield. ^1^H NMR (CDCl_3_) δ 7.26 (s, 1H), 7.04 (s, 1H), 3.77 (br s, 1H), 3.38 (br s, 3H), 2.54 (s, 3H), 2.34 (s, 3H). Found [M+H]=195.

This Weinreb amide was similarly converted into 3-(dimethylamino)-1-(4,6-dimethylpyridin-2-yl)propan-1-one in 98% yield. ^1^H NMR (CDCl_3_) δ 7.64 (s, 1H), 7.11 (s, 1H), 3.36 (t, *J* = 7.1 Hz, 2H), 2.74 (t, *J* = 7.1 Hz, 2H), 2.54 (s, 3H), 2.34 (s, 3H), 2.27 (s, 6H). Found: [M+H]=207.

**Class E: 3-(Dimethylamino)-1-(3,5-dimethylpyridin-2-yl)propan-1-one**

Similarly was prepared, from 3,5-dimethylpicolinic acid, N-methoxy-N,3,5-trimethylpicolinamide in 23% yield. ^1^H NMR (CDCl_3_) δ 8.25 (d, *J* = 0.6 Hz, 1H), 7.36 (s, 1H), 3.56 (s, 3H), 3.37 (s, 3H), 2.33 (s, 3H), 2.31 (s, 3H). Found: [M+H]=195.

This Weinreb amide was similarly converted into 3-(dimethylamino)-1-(3,5-dimethylpyridin-2-yl)propan-1-one in 99% yield. ^1^H NMR (CDCl_3_) δ 8.32 (dd, *J* = 1.3, 0.5 Hz, 1H), 7.37 (dd, *J* = 1.3, 0.7 Hz, 1H), 3.35 (t, *J* = 7.2 Hz, 2H), 2.73 (t, *J* = 7.2 Hz, 2H), 2.54 (s, 3H), 2.36 (s, 3H), 2.27 (s, 6H). Found: [M+H]=207.

**Class F: 3-(Dimethylamino)-1-(6-methoxypyridin-2-yl)propan-1-one**

Similarly was prepared, starting from 6-methoxypicolinic acid, *N*,6-dimethoxy-*N*-methylpicolinamide in 92% yield. ^1^H NMR (CD_3_SOCD_3_) δ 7.86 (dd, *J* = 8.3, 7.3 Hz, 1H), 7.66 (dd, *J* = 7.3, 0.7 Hz, 1H), 7.05 (dd, *J* = 8.3, 0.7 Hz, 1H), 3.90 (s, 3H), 3.28 (bs, 3H). Found: [M+H]=197.

The Weinreb amide was similarly converted into 3-(dimethylamino)-1-(6-methoxypyridin-2-yl)propan-1-one in 69% yield. ^1^H NMR (CDCl_3_) δ 7.69 (t, *J* = 7.3 Hz, 1H), 7.64 (dd, *J* = 7.3, 1.0 Hz, 1H), 6.93 (dd, *J* = 8.0, 1.0 Hz, 1H), 3.99 (s, 3H), 3.36 (t, *J* = 7.2 Hz, 2H), 2.77 (t, *J* = 7.2 Hz, 2H), 2.29 (s, 3H). Found: [M+H]=209

**Class G: 3-(Dimethylamino)-1-(4-methoxypyridin-2-yl)propan-1-one**

Similarly was prepared, starting from 4-methoxypicolinic acid, N,4-dimethoxy-N-methylpicolinamide in 72% yield. ^1^H NMR (CDCl_3_) δ 8.42 (d, *J* = 5.7 Hz, 1H), 7.19 (s, 1H), 6.87 (dd, *J* = 5.7, 2.6 Hz, 1H), 3.89 (s, 3H), 3.77 (s, 3H), 3.41 (s, 3H). Found: [M+H]=197

This Weinreb amide was similarly converted into 3-(dimethylamino)-1-(4-methoxypyridin-2-yl)propan-1-one in 99% yield. ^1^H NMR (CDCl_3_) δ 8.48 (d, *J* = 5.6 Hz, 1H), 7.57 (d, *J* = 2.6 Hz, 1H), 6.97 (dd, *J* = 5.6, 2.6 Hz, 1H), 3.90 (s, 3H), 3.38 (t, *J* = 7.2 Hz, 2H), 2.77 (t, *J* = 7.2 Hz, 2H), 2.28 (s, 6H). Found: [M+H]=209

**Class H: 3-(Dimethylamino)-1-(2-methylpyridin-3-yl)propan-1-one**

Similarly was prepared, starting from 2-methylnicotinic acid, N-methoxy-N,2-dimethylnicotinamide in 83% yield. ^1^H NMR (CDCl_3_) δ 8.55 (dd, *J* = 4.9, 1.8 Hz, 1H), 7.60 (dd, *J* = 7.7, 1.8 Hz, 1H), 7.16 (dd, *J* = 7.7, 4.9 Hz, 1H), 3.46 (br s, 3H), 3.36 (br s, 3H), 2.57 (s, 3H). Found: [M=H]=181.

This Weinreb amide was similarly converted into 3-(dimethylamino)-1-(2-methylpyridin-3-yl)propan-1-one in 75% yield. ^1^H NMR (CDCl_3_) δ 8.58 (dd, *J* = 4.9, 1.7 Hz, 1H), 7.90 (dd, *J* = 7.8, 1.7 Hz, 1H), 7.22 (dd, *J* = 7.8, 4.9 Hz, 1H), 3.05 (t, *J* = 7.0 Hz, 2H), 2.71 (t, *J* = 7.0 Hz, 2H), 2.71 (s, 3H), 2.25 (s, 6H). Found: [M+H]=193.

**Class I: 3-(Dimethylamino)-1-(4-methylpyridin-3-yl)propan-1-one**

Similarly was prepared, starting from4-methylnicotinic acid, N-methoxy-N,5-dimethylnicotinamide in 95% yield. ^1^H NMR (CDCl_3_) δ 8.87 (d, *J* = 1.8 Hz, 1H), 7.95 (dd, *J* = 8.0, 2.2 Hz, 1H), 7.21 (d, *J* = 8.0 Hz, 1H), 3.56 (s, 3H), 3.38 (s, 3H), 2.61 (s, 3H). Found: [M+H]=181

This Weinreb amide was similarly converted into 3-(dimethylamino)-1-(4-methylpyridin-3-yl)propan-1-one in 97% yield. ^1^H NMR (CDCl_3_) δ 9.06 (d, *J* = 2.0 Hz, 1H), 8.13 (dd, *J* = 8.1, 2.3 Hz, 1H), 7.27 (d, *J* = 8.1 Hz, 1H), 3.14 (t, *J* = 7.2 Hz, 2H), 2.76 (t, *J* = 7.2 Hz, 2H), 2.63 (s, 3H), 2.29 (s, 6H). Found: [M+H]=193

**Class J: 3-(Dimethylamino)-1-(5-methylpyridin-3-yl)propan-1-one**

Similarly was prepared, starting from 5-methylnicotinic acid, N-methoxy-N,5-dimethylnicotinamide in 87% yield. ^1^H NMR (CDCl_3_) δ 8.75 (d, *J* = 1.7 Hz, 1H), 8.52 (d, *J* = 1.7 Hz, 1H), 8.03 (m, 1H), 3.57 (s, 3H), 2.85 (s, 3H), 3.17 (s, 3H). Found: [M+H]=181.

This Weinreb amide was similarly converted into 3-(dimethylamino)-1-(5-methylpyridin-3-yl)propan-1-one in 93% yield. ^1^H NMR (CDCl_3_) δ 8.98 (d, *J* = 1.9 Hz, 1H), 8.61 (d, *J* = 1.6 Hz, 1H), 8.03 (m, 1H), 3.15 (t, *J* = 7.0 Hz, 2H), 2.77 (t, *J* = 7.0 Hz, 2H), 2.29 (s, 6H). Found: [M+H]=193.

**Class K: 3-(Dimethylamino)-1-(2,6-dimethylpyridin-3-yl)propan-1-one**

Similarly was prepared, starting from 2,6-dimethylnicotinic acid, N-methoxy-N,2,6-trimethylnicotinamide in 66% yield. ^1^H NMR (CDCl_3_) δ 7.50 (d, *J* = 7.8 Hz, 1H), 7.02 (d, *J* = 7.8 Hz, 1H), 3.48 (s, 3H), 3.34 (s, 3H), 2.55 (s, 3H), 2.54 (s, 3H). Found: [M+H]=195

This Weinreb amide was similarly converted into 3-(dimethylamino)-1-(2,6-dimethylpyridin-3-yl)propan-1-one in 99% yield. ^1^H NMR (CDCl_3_) δ 7.84 (d, *J* = 8.0 Hz, 1H), 7.07 (d, *J* = 8.0 Hz, 1H), 3.04 (t, *J* = 7.2 Hz, 2H), 2.70 (t, *J* = 7.2 Hz, 2H), 2.69 (s, 3H), 2.57 (s, 3H), 2.26 (s, 6H). Found: [M+H]=207

**Class L: 3-(Dimethylamino)-1-(5,6-dimethylpyridin-3-yl)propan-1-one**

Similarly was prepared, starting from 5,6-dimethylnicotinic acid, N-methoxy-N,5,6-trimethylnicotinamide in 92% yield. ^1^H NMR (CDCl_3_) δ 8.69 (d, *J* = 1.8 Hz, 1H), 7.76 (d, *J* = 1.6 Hz, 1H), 3.57 (s, 3H), 3.37 (s, 3H), 2.55 (s, 3H), 2.32 (s, 3H). Found: [M+H]=195

This Weinreb amide was similarly converted into 3-(dimethylamino)-1-(5,6-dimethylpyridin-3-yl)propan-1-one in 98% yield. ^1^H NMR (CDCl_3_) δ 8.89 (d, *J* = 2.1 Hz, 1H), 7.95 (d, *J* = 1.6 Hz, 1H), 3.13 (t, *J* = 7.3 Hz, 2H), 2.76 (t, *J* = 7.3 Hz, 2H), 2.57 (s, 3H), 2.35 (s, 3H), 2.29 (s, 6H). Found: [M+H]=207

**Class M: 3-(Dimethylamino)-1-(4,6-dimethylpyridin-3-yl)propan-1-one**

Similarly was prepared, starting from 4,6-dimethylnicotinic acid, N-methoxy-N,4,6-trimethylnicotinamide in 93% yield. ^1^H NMR (CDCl_3_) δ 8.40 (s, 1H), 7.03 (s, 1H), 3.50 (s, 3H), 3.48 (s, 3H), 2.54 (s, 3H), 2.32 (s, 3H). Found: [M+H]=195

This Weinreb amide was similarly converted into 3-(dimethylamino)-1-(4,6-dimethylpyridin-3-yl)propan-1-one in 99% yield. ^1^H NMR (CDCl_3_) δ 8.82 (s, 1H), 7.05 (s, 1H), 3.09 (t, *J* = 7.2 Hz, 2H), 2.72 (t, *J* = 7.2 Hz, 2H), 2.55 (s, 3H), 2.50 (s, 3H), 2.26 (s, 6H). Found: [M+H]=207

**Class N: 3-(Dimethylamino)-1-(2-methoxypyridin-3-yl)propan-1-one**

Similarly was prepared, starting from 2-methoxynicotinic acid, *N*,2-dimethoxy-*N*-methylnicotinamide in 78% yield. ^1^H NMR (CDCl_3_) δ 8.22 (dd, *J* = 5.0, 1.8 Hz, 1H), 7.60 (bd, *J* = 6.3 Hz, 1H), 6.93 (dd, *J* = 7.2, 5.0 Hz, 1H), 3.99 (s, 3H), 3.54 (bs, 3H), 3.33 (bs, 3H). Found: [M+H]=197.

The Weinreb amide was similarly converted to 3-(dimethylamino)-1-(2-methoxypyridin-3-yl)propan-1-one in 95% yield. ^1^H NMR (CDCl_3_) δ 8.30 (dd, *J* = 4.8, 2.0 Hz, 1H), 8.08 (dd, *J* = 7.6, 2.0 Hz, 1H), 6.98 (dd, *J* = 7.5, 4.8 Hz, 1H), 4.05 (s, 3H), 3.21 (t, *J* = 7.1 Hz, 2H), 2.71 (t, *J* = 7.1 Hz, 2H), 2.27 (s, 6H). Found: [M+H]=209.

**Class O: 3-(Dimethylamino)-1-(6-methoxypyridin-3-yl)propan-1-one**

Similarly was prepared, starting from 6-methoxynicotinic acid, N,6-dimethoxy-N-methylnicotinamide in 83% yield. ^1^H NMR (CDCl_3_) δ 8.65 (dd, *J* = 2.4, 0.5 Hz, 1H), 8.00 (dd, *J* = 8.7, 2.4 Hz, 1H), 6.76 (dd, *J* = 8.7, 0.7 Hz, 1H), 3.99 (s, 3H), 3.58 (s, 3H), 3.38 (s, 3H). Found: [M+H]=197

This Weinreb amide was similarly converted into 3-(dimethylamino)-1-(6-methoxypyridin-3-yl)propan-1-one in 99% yield. ^1^H NMR (CDCl_3_) δ 8.81 (dd, *J* = 2.3, 0.4 Hz, 1H), 8.15 (dd, *J* = 8.7, 2.4 Hz, 1H), 6.79 (dd, *J* = 8.7, 0.6 Hz, 1H), 4.00 (s, 3H), 3.09 (t, *J* = 7.3 Hz, 2H), 2.75 (t, *J* = 7.3 Hz, 2H), 2.29 (s, 6H). Found: [M+H]=209

**Class P: 1-(2,5-Dimethoxypyridin-3-yl)-3-(dimethylamino)propan-1-one**

Similarly was prepared, starting from 2,5-dimethoxynicotinic acid, N,2,5-trimethoxy-N-methylnicotinamide in 61% yield. ^1^H NMR (CDCl_3_) δ 7.87 (br s, 1H), 7.24 (br s, 1H), 3.94 (s, 3H), 8.82 (s, 3H), 3.56 (br s, 3H), 3.32 (br s, 3H). Found: [M+H]=227.

This Weinreb amide was similarly converted into 1-(2,5-dimethoxypyridin-3-yl)-3-(dimethylamino)propan-1-one in 90% yield. ^1^H NMR (CDCl_3_) δ 8.00 (d, *J* = 3.2 Hz, 1H), 7.69 (d, *J* = 3.2 Hz, 1H), 4.01 (s, 3H), 3.83 (s, 3H), 3.22 (t, *J* = 7.1 Hz, 2H), 2.70 (t, *J* = 7.1 Hz, 2H), 2.27 (s, 6H). Found: [M+H]=239.

**Class Q: 1-(5,6-Dimethoxypyridin-3-yl)-3-(dimethylamino)propan-1-one**

Similarly was prepared, starting from 5,6-dimethoxynicotinic acid, N,5,6-trimethoxy-N-methylnicotinamide in 85% yield. ^1^H NMR (CDCl_3_) δ 8.26 (d, J = 1.9 Hz, 1H), 7.49 (d, J = 1.9 Hz, 1H), 4.11 (s, 3H), 3.91 (s, 3H), 3.61 (s, 3H), 3.39 (s, 3H). Found: [M+H]=227.

The Weinreb amide was similarly converted to 1-(5,6-dimethoxypyridin-3-yl)-3-(dimethylamino)prpoan-1-one in 32% yield. ^1^H NMR (CDCl_3_) δ 8.40 (d, J = 1.9 Hz, 1H), 7.61 (d, J = 1.9 Hz, 1H), 4.09 (s, 3H), 3.92 (s, 3H), 3.11 (t, J = 7.1 Hz, 2H), 2.77 (t, J = 7.1 Hz, 2H), 2.30 (s, 6H). Found: [M+H]=239.

**Class R: 3-(Dimethylamino)-1-(2,4,5-trimethoxypyridin-3-yl)propan-1-one**

Similarly was prepared, starting from 2,4,5-trimethoxynicotinic acid, N,2,4,5-tetramethoxy-N-methylnicotinamide in 94% yield. ^1^H NMR (CDCl_3_) δ 7.20 (s, 1H), 4.03 (s, 3H), 3.94 (s, 3H), 3.83 (s, 3H), 3.64 (br s, 3H), 3.30 (s, 3H). Found [M+H]=257.

This Weinreb amide was similarly converted into 3-(dimethylamino)-1-(2,4,5-trimethoxypyridin-3-yl)propan-1-one in 92% yield. . ^1^H NMR (CDCl_3_) δ 7.71 (s, 1H), 4.07 (s, 3H), 4.02 (s, 3H), 3.86 (s, 3H), 3.19 (t, *J* = 7.1 Hz, 2H), 2.70 (t, *J* = 7.1 Hz, 2H), 2.29 (s, 6H). Found: [M+H]=269.

**Class S: 3-(Dimethylamino)-1-(3-methylpyridin-4-yl)propan-1-one**

Similarly was prepared, starting from 3-methylisonicotinic acid, N-methoxy-N,3-dimethylisonicotinamide in 90% yield. ^1^H NMR (CDCl_3_) δ 8.51 (s, 1H), 8.49 (d, *J* = 4.9 Hz, 1H), 7.18 (d, *J* = 4.9 Hz, 1H)), 3.38 (s, 3H), 3.37 (s, 3H), 2.32 (s, 3H). Found: [M+H]=181

This Weinreb amide was similarly converted into 3-(dimethylamino)-1-(3-methylpyridin-4-yl)propan-1-one in 31% yield. ^1^H NMR (CDCl_3_) δ 8.57 (dd, *J* = 5.0, 0.4 Hz, 1H), 8.55 (s, 1H), 7.37 (d, *J* = 5.0 Hz, 1H), 3.03 (t, *J* = 7.1 Hz, 2H), 2.69 (t, *J* = 7.1 Hz, 2H), 2.43 (s, 3H), 2.24 (s, 6H). Found: [M+H]=193

**Class T: 3-(dimethylamino)-1-(2-methylpyridin-4-yl)propan-1-one**

Similarly was prepared, starting from 2-methylisonicotinic acid, N-methoxy-N,2-dimethylisonicotinamide in 79% yield. ^1^H NMR (CDCl_3_) δ 8.58 (dd, *J* = 5.0, 0.5 Hz, 1H), 7.37 (s, 1H), 7.30 (d, *J* = 5.0 Hz, 1H), 3.55 (s, 3H), 3.36 (s, 3H), 2.61 (s, 3H). Found: [M+H]=181

This Weinreb amide was similarly converted into 3-(dimethylamino)-1-(2-methylpyridin-4-yl)propan-1-one in 99% yield. ^1^H NMR (CDCl_3_) δ 8.68 (dd, *J* = 5.1, 0.4 Hz, 1H), 7.59 (s, 1H), 7.52 (dd, *J* = 5.1, 1.0 Hz, 1H), 3.12 (t, *J* = 7.2 Hz, 2H), 2.75 (t, *J* = 7.2 Hz, 2H), 2.64 (s, 3H), 2.28 (s, 6H). Found: [M+H]=193

**Class U: 3-(Dimethylamino)-1-(2,6-dimethylpyridin-4-yl)propan-1-one**

Similarly was prepared, starting from 2,6-dimethylisonicotinic acid, N-methoxy-N,2,6-trimethylisonicotinamide in 49% yield. ^1^H NMR (CDCl_3_) δ 7.15 (s, 2H), 3.56 (s, 3H), 3.35 (s, 3H), 2.57 (s, 6H). Found: [M+H]=195

This Weinreb amide was similarly converted into 3-(dimethylamino)-1-(2,6-dimethylpyridin-4-yl)propan-1-one in 99% yield. ^1^H NMR (CDCl_3_) δ 7.38 (s, 2H), 3.10 (t, *J* = 7.2 Hz, 2H), 2.74 (t, *J* = 7.2 Hz, 2H), 2.61 (s, 6H), 2.28 (s, 6H). Found: [M+H]=207

**Class V:** **1-(2,6-Diethoxypyridin-4-yl)-3-(dimethylamino)propan-1-one**

Similarly was prepared, starting from 2,6-biethoxyisonicotinic acid, 2,6-diethoxy-N-methoxy-N-methylisonicotinamide in 69% yield. ^1^H NMR (CDCl_3_) δ 6.43 (s, 2H), 4.33 (q, *J* = 7.1 Hz, 2H), 3.59 (br s, 3H), 3.32 (s, 3H), 1.39 (t, *J* = 7.1 Hz, 3H). Found: [M+H]=255

This Weinreb amide was similarly converted into 3-(dimethylamino)-1-(2,6-dimethylpyridin-4-yl)propan-1-one in 96% yield. ^1^H NMR (CDCl_3_) δ 6.71 (s, 2H), 4.34 (q, *J* = 7.1 Hz, 2H), 3.05 (t, *J* = 7.0 Hz, 2H), 2.72 (t, *J* = 7.0 Hz, 2H), 2.26 (s, 6H), 1.40 (t, *J* = 7.0 Hz, 3H). Found: [M+H]=267

**Class W: 1-(2,5-Dimethoxypyridin-4-yl)-3-(dimethylamino)propan-1-one**

Similarly was prepared, from 2,5-dimethoxyisonicotinic acid, N,2,5-trimethoxy-N-methylisonicotinamide in 41% yield. ^1^H NMR (CDCl_3_) δ 7.82 (s, 1H), 6.60 (br s, 1H), 3.90 (s, 3H), 3.89 (s, 3H), 3.51 (br s, 1H), 3.35 (br s, 3H). Found: [M+H]=227.

This Weinreb amide was similarly converted into 1-(2,5-dimethoxypyridin-4-yl)-3-(dimethylamino)propan-1-one in 67% yield. ^1^H NMR (CDCl_3_) δ 7.89 (s, 1H), 6.86 (d, *J* = 0.4 Hz, 1H), 3.91 (s, 3H), 3.90 (s, 3H), 3.10 (t, *J* = 7.1 Hz, 2H), 2.67 (t, *J* = 7.1 Hz, 2H), 2.24 (s, 6H). Found: [M+H]=239.

**Representative synthesis of the 6-bromo analogues of Table 2.**

**1-(7-Bromo-3-methoxynaphthalen-2-yl)-1-(2,3-dimethoxyphenyl)-4-(dimethylamino)-2-(2,6-dimethylpyridin-4-yl)butan-2-ol (56)**

A solution of dry diisopropylamine (1.30 ml, 9.28 mmol) in dry THF (10 ml) was cooled to -40 ^o^C under an atmosphere of dry nitrogen. *N*-Butyllithium (4.65 ml of a 2.0 N solution in cyclohexane, 9.28 mmol) was added dropwise, then stirring was continued for a further 15 min. The solution was cooled to -70 to -78 ^o^C and a solution of 6-bromo-3-(2,3-dimethoxybenzyl)-2-methoxyquinoline^S2^ (3.00 g, 7.73 mmol) in dry THF (6 ml) was added dropwise. The resulting purple solution was stirred at this temperature for 90 min. A solution of 3-(dimethylamino)-1-(2,6-dimethylpyridin-4-yl)propan-1-one (class U Mannich base) (1.75 g, 8.50 mmol) in THF (6 ml) was then added dropwise and the mixture was stirred at this temperature for 5 h. Glacial acetic acid (0.70 ml) was added in one portion and the mixture was allowed to warm to room temperature. Water was added and the mixture was extracted with ethyl acetate. The extract was washed with water and dried over sodium sulfate. Removal of the solvent under reduced pressure left an oil, which was chromatographed on silica. Column chromatography with DCM gave fore fractions, followed by isomer A of **56** (1.90 g, 41%). Elution with DCM:MeOH (92:8) gave isomer B of **9** (1.01 g, 22%).

Isomer A, white solid. ^1^H NMR (CDCl_3_, 400 MHz) δ 8.21 (s, 1H), 7.83 (d, J = 2.2 Hz, 1H), 7.71-7.66 (m, 2H), 7.60 (dd, J = 8.9, 2.2 Hz, 1H), 7.20-7.13 (m, 3H), 6.83 (t, J = 8.1 Hz, 1H), 6.59 (dd, J = 8.2, 1.4 Hz, 1H), 5.60 (s, 1H), 4.23 (s, 3H), 3.68 (s, 3H), 3.47 (s, 3H), 2.47 (s, 6H), 2.28-2.20 (m, 1H), 2.00-1.96 (m, 1H), 1.99 (s, 6H), 1.85-1.80 (m, 2H). Found: [M+H]= 594.6.

Isomer B, white solid. ^1^H NMR (CDCl_3_, 400 MHz) δ 8.65 (s, 1H), 7.81 (d, J = 2.1 Hz, 1H), 7.57 (dd, J = 8.9, 2.0 Hz, 1H), 7.62 (d, J = 8.9 Hz, 1H), 7.28-7.21 (m, 4H), 7.05 (t, J = 8.0 Hz, 1H), 6.87 (d, J = 8.6 Hz, 1H), 4.95 (s, 1H), 3.99 (s, 3H), 3.90 (s, 3H), 3.90 (s, 3H), 2.23- 2.18 (m, 2H), 2.43 (s, 6H), 2.03 (s, 6H), 1.95-1.80 (m, 2H). Found: [M+H]= 594.6.

Each coupled product was resolved into its four optical isomers using preparative chiral HPLC at BioDuro LLC (Beijing).

The other 6-bromo analogues of Table 1 were prepared similarly:

**Representative synthesis of the 6-cyano analogues of Table 2.**

**7-(1-(2,3-Dimethoxyphenyl)-4-(dimethylamino)-2-(2,6-dimethylpyridin-4-yl)-2-hydroxybutyl)-6-methoxy-2-naphthonitrile (57)**

A solution of **56** (1.62 g, 2.73 mmol) in DMF (10 mL, anhydrous) was purged with nitrogen and heated to 55 °C for 10 min. Tri(*o*-tolyl)phosphine (0.125 g, 0.41 mmol), zinc dust (0.018 g, 0.273 mmol) and tris(dibenzylideneacetone)dipalladium(0) (0.188 g, 0.205 mmol) were then added, and the reaction was again purged with nitrogen and heated for another 10 min at 55 °C. Zinc cyanide (0.177 g, 1.50 mmol) was then added and the reaction mixture was heated to 65 °C for 4 hours. The reaction was diluted with water and extracted with EtOAc three times. The organic layer was washed with brine three times, dried and evaporated. Column chromatography with DCM followed afforded isomer A of **57** (0.65 g, 44%) followed by isomer B of **57** (0.46 g, 31%) as white solids.

Isomer A, white solid. ^1^H NMR (CDCl_3_, 400 MHz) δ 8.33 (s, 1H), 8.16 (s, 1H), 8.06 (d, J = 1.8 Hz, 1H), 7.85 (d, J = 8.6 Hz, 1H), 7.73-6.98 (m, 2H), 7.21-7.11 (m, 2H), 6.84 (t, J = 8.1 Hz, 1H), 6.60 (dd, J = 8.2, 1.4 Hz, 1H), 5.60 (s, 1H), 4.28 (s, 3H), 3.68 (s, 3H), 3.49 (s, 3H), 2.47 (s, 6H), 2.21-2.13 (m, 1H), 2.02-1.96 (m, 1H), 1.95 (s, 6H), 1.85-1.72 (m, 2H). Found: [M+H]= 542.4.

Isomer B, white solid. ^1^H NMR (CDCl_3_, 400 MHz) δ 8.79 (s, 1H), 8.21 (s, 1H), 8.03 (d, J = 1.7 Hz, 1H), 7.68 (d, J = 8.6 Hz, 1H), 7.62 (dd, J = 8.6, 1.8 Hz, 1H), 7.37 (dd, J = 8.0, 1.4 Hz, 1H), 7.13-7.06 (m, 2H), 7.00 (t, J = 8.0 Hz, 1H), 6.82 (dd, J = 8.0, 1.4 Hz, 1H), 5.54 (s, 1H), 4.01 (s, 3H), 3.90 (s, 3H), 3.90 (s, 3H), 2.43 (s, 6H), 2.20-2.12 (m, 1H), 2.02 (s, 6H), 2.01-1.93 (m, 2H), 1.74-1.68 (m, 1H). Found: [M+H]=542.4.

In each case, the coupled 6-cyano product was then resolved into its four optical isomers using preparative chiral HPLC at BioDuro LLC (Beijing).

The other 6-cyano analogues of Table 1 were prepared similarly:

**References**

S1. Guillemont JEG, Meyer C, Poncelet A, Bourdrez X, Andries K. Fut. Med. Chem. 2011; 3: 1345-1360.

S2. Tong AST, Choi PJ, Blaser A, Sutherland HS, Tsang SKY, Guillemont J, Motte M, Cooper CB, Andries K, Van den Broeck W, Franzblau SG, Upton MA, Denny WA, Palmer BD, Conole D. 6-Cyano analogues of bedaquiline as less lipophilic and potentially safer diarylquinolines for tuberculosis. ACS Med. Chem. Lett. 2017; 8: 1019-1024.

S3. Choi, PJ, Sutherland HS, Tong AST, Blaser A, Franzblau SG, Cooper, CB, Lotlikar MA, Upton MA, Guillemont J, Motte M, Queguiner L, Andries K, Van den Broeck W, Denny WA, Palmer BS3. D. Synthesis and evaluation of analogues of the tuberculosis drug bedaquiline containing heterocyclic B-ring units. Bioorg. Med. Chem. Lett. 2017; 27: 5190-5196.

S4. Sutherland HS, Tong AST, Choi PJ, Conole D, Blaser A, Franzblau SG, Cooper CB, Upton AM, Lotlikar MU, Denny WA, Palmer BD. Structure-activity relationships for analogs of the tuberculosis drug bedaquiline with the naphthalene unit replaced by bicyclic heterocycles. Bioorg. Med. Chem. 2018; 26: 1797-1809.
